# PROTAC-Induced Glycogen Synthase Kinase 3β Degradation as a Potential Therapeutic Strategy for Alzheimer’s Disease

**DOI:** 10.1021/acschemneuro.3c00096

**Published:** 2023-05-23

**Authors:** Melissa Guardigni, Letizia Pruccoli, Alan Santini, Angela De Simone, Matteo Bersani, Francesca Spyrakis, Flavia Frabetti, Elisa Uliassi, Vincenza Andrisano, Barbara Pagliarani, Paula Fernández-Gómez, Valle Palomo, Maria Laura Bolognesi, Andrea Tarozzi, Andrea Milelli

**Affiliations:** † Department for Life Quality Studies, Alma Mater Studiorum-University of Bologna, Corso d’Augusto 237, 47921 Rimini, Italy; ‡ Department of Drug Science and Technology, University of Turin, Via Pietro Giuria 9, 10125 Torino, Italy; § Department of Medical and Surgical Sciences, Alma Mater Studiorum-University of Bologna, Via Belmeloro 8, 40126 Bologna, Italy; ∥ Department of Pharmacy and Biotechnology, Alma Mater Studiorum-University of Bologna, Via Belmeloro 6, 40126 Bologna, Italy; ⊥ Instituto Madrileño de Estudios Avanzados en Nanociencia (IMDEA-Nanociencia), C/Faraday 9, 28049 Madrid, Spain; # Centro de Investigación Biomédica en Red de Enfermedades Neurodegenerativas (CIBERNED), Instituto de Salud Carlos III, Av. de Monforte de Lemos, 5, 28029 Madrid, Spain

**Keywords:** proteolysis targeting chimeras, glycogen synthase kinase 3β, Alzheimer’s disease, chemical knockdown, protein degradation

## Abstract

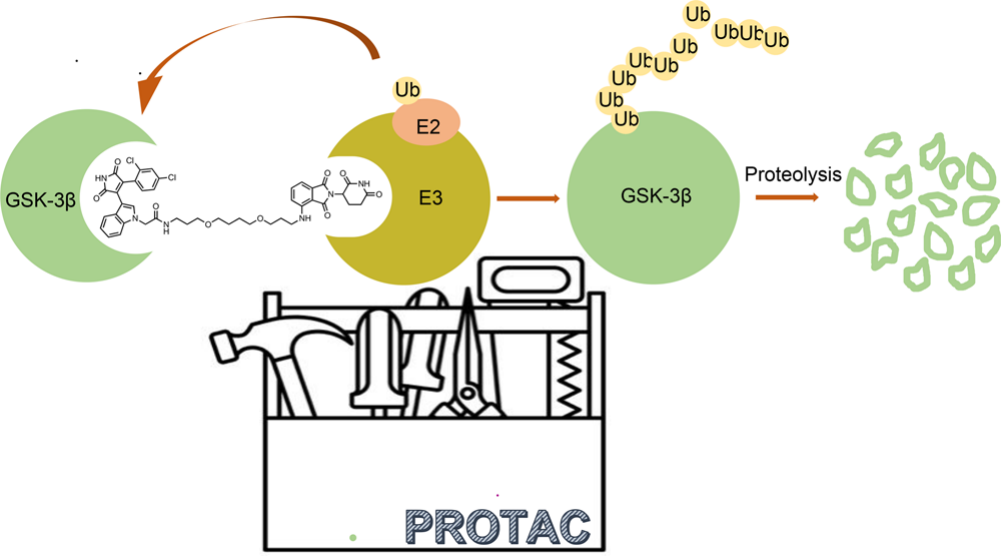

Glycogen synthase kinase 3β (GSK-3β) is a serine/threonine kinase and an attractive therapeutic target for Alzheimer’s disease. Based on proteolysis-targeting chimera (PROTAC) technology, a small set of novel GSK-3β degraders was designed and synthesized by linking two different GSK-3β inhibitors, SB-216763 and tideglusib, to pomalidomide, as E3 recruiting element, through linkers of different lengths. Compound **1** emerged as the most effective PROTAC being nontoxic up to 20 μM to neuronal cells and already able to degrade GSK-3β starting from 0.5 μM in a dose-dependent manner. PROTAC **1** significantly reduced the neurotoxicity induced by Aβ_25–35_ peptide and CuSO_4_ in SH-SY5Y cells in a dose-dependent manner. Based on its encouraging features, PROTAC **1** may serve as a starting point to develop new GSK-3β degraders as potential therapeutic agents.

## Introduction

Glycogen synthase kinase 3β (GSK-3β) is a highly conserved serine/threonine kinase ubiquitously expressed and constitutively active.^1^ Being involved in crucial signaling pathways (such as PI3K, Wnt, Hedgehog, Notch) and regulating a wide spectrum of cellular functions, it is highly implicated in a series of diseases such as cancer, diabetes, and inflammatory, immune, and neurological disorders.^1^ Regarding neurological conditions, GSK-3β controls a multitude of central nervous system (CNS)-specific signaling pathways related to development, metabolic homeostasis, neuronal growth, and differentiation,^2^ and it is found to be hyperactivated in the brain of Alzheimer’s disease (AD) patients.^3^ Compelling evidence supports GSK-3β as the main kinase involved in AD pathology, being implicated in tau- and Aβ-mediated toxicities as well as in oxidative stress, inflammation, memory formation, and synaptic plasticity.^4^ Furthermore, GSK-3β is networked with several other factors involved in AD.^5^ In light of this, GSK-3β represents a promising drug target and a multitude of inhibitors have been developed, some of which have reached clinical studies.^6^ Based on the mechanism of action, such inhibitors can be categorized into ATP competitive and non-ATP competitive inhibitors.^7^

Recently, beyond classical target inhibition, a new paradigm based on so-called proteolysis targeting chimeras (PROTACs) is in the spotlight.^8^ This revolutionary modality uses small-molecule PROTACs to control protein levels rather than modulating its function. Indeed, PROTACs do not inhibit a given protein of interest (POI) but instead induce its removal by binding to it and by harnessing the cell disposal ubiquitin–proteasome system (UPS). Based on this mechanism of action, PROTACs catalytically remove different quantities of proteins through multiple rounds of activity and trigger potent effects even at low doses. As such, many issues associated with classical small molecule inhibitors, such as drug resistance and adverse effects, could be avoided.^9,10^ Since the first report almost 20 years ago, more than 1000 different PROTACs have been described, and some of them have entered clinical trials.^11^

Last year, two research groups independently reported about PROTACs targeting GSK-3β.^12,13^ These two PROTACs turned out to be able to induce GSK-3β degradation in cells, and one of them was also effective in an AD mouse model.^12^

**Figure 1:**
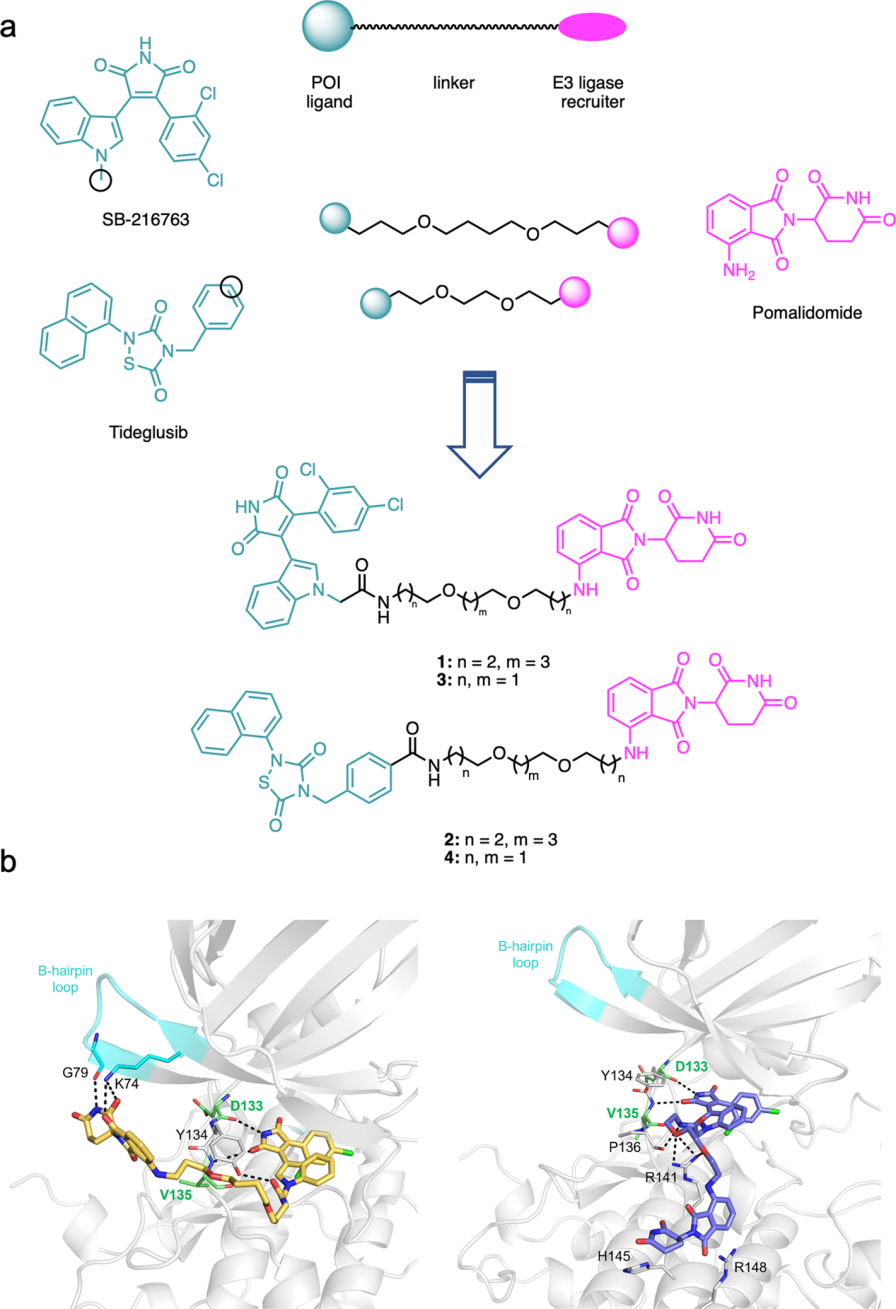
(a) Design strategy leading to GSK-3β-directed PROTAC **1**–**4**. Black circles represent tethering points. (b) MD-extracted structure of the **1**–GSK-3β (left) and **3**–GSK-3β (right) complexes showing H-bond contacts with kinase residues.

Owing to the availability of an arsenal of GSK-3β inhibitors and our interest in GSK-3β for AD,^5,14,15^ we sought to develop new PROTACs characterized by different and previously unexplored GSK-3β recruiting elements. Particularly, we were interested to evaluate whether any difference could be observed between an ATP competitive and non-ATP competitive GSK-3β engagement. Considering this insight, in this Letter, we report the preliminary design, synthesis, and evaluation of these novel GSK-3β-directed PROTACs (Figure 1a).

## Results and Discussion

### Drug Design

From a medicinal chemistry point of view, PROTACs are heterobifunctional molecules consisting of a POI-binding ligand, connected via a linker to a recruitment moiety for an E3 ubiquitin ligase, leading to polyubiquitination and degradation of the POI.^16^ To develop such PROTACs we focused our attention on compounds SB-216763 and tideglusib as GSK-3β recruiting elements (Figure 1). We selected these compounds because they are characterized by two different mechanisms of inhibition. SB-216763 is the prototype of reversible maleimide-based ATP-competitive inhibitors able to block GSK-3β with high selectivity,^17^ while tideglusib selectively inhibits GSK-3β, with a non-competitive inhibition pattern with respect to ATP.^18^ Analyzing the structure–activity relationships (SAR) of SB-216763, it turned out that the indolyl nitrogen may be substituted with groups bulkier than methyl without significant activity reduction.^7^ Similarly, we observed that a chain may be introduced at the phenyl group para-position of tideglusib, by means of amide-bond connection, without significant loss of the inhibitory activity (unpublished results).

Therefore, we identified these two positions, i.e., the indolyl nitrogen of SB-216763 and the para-position of the phenyl ring of tideglusib, as the tethering site to the E3 ligase recruiting element. As cereblon (CRBN) E3 ligase recruiter, pomalidomide was selected. Since the degrading potential of PROTACs depends on their ability to form a ternary complex with the POI and the ligase, we hypothesized the initial use of two linkers of different length, as 3–4–3 and 2–2–2-poly(ethylene glycol), designing compounds **1**–**4** (Figure 1a). We selected PEG linkers since they are by far the most common motifs incorporated into PROTACs due to their favorable properties, in terms of synthetic accessibility, flexibility, availability, and physicochemical profile.^19^ To support such choice, we performed computational analysis to assess whether the chosen linkers were able to project the E3 ligase-binding element outside the POI binding site.

**Figure 2:**
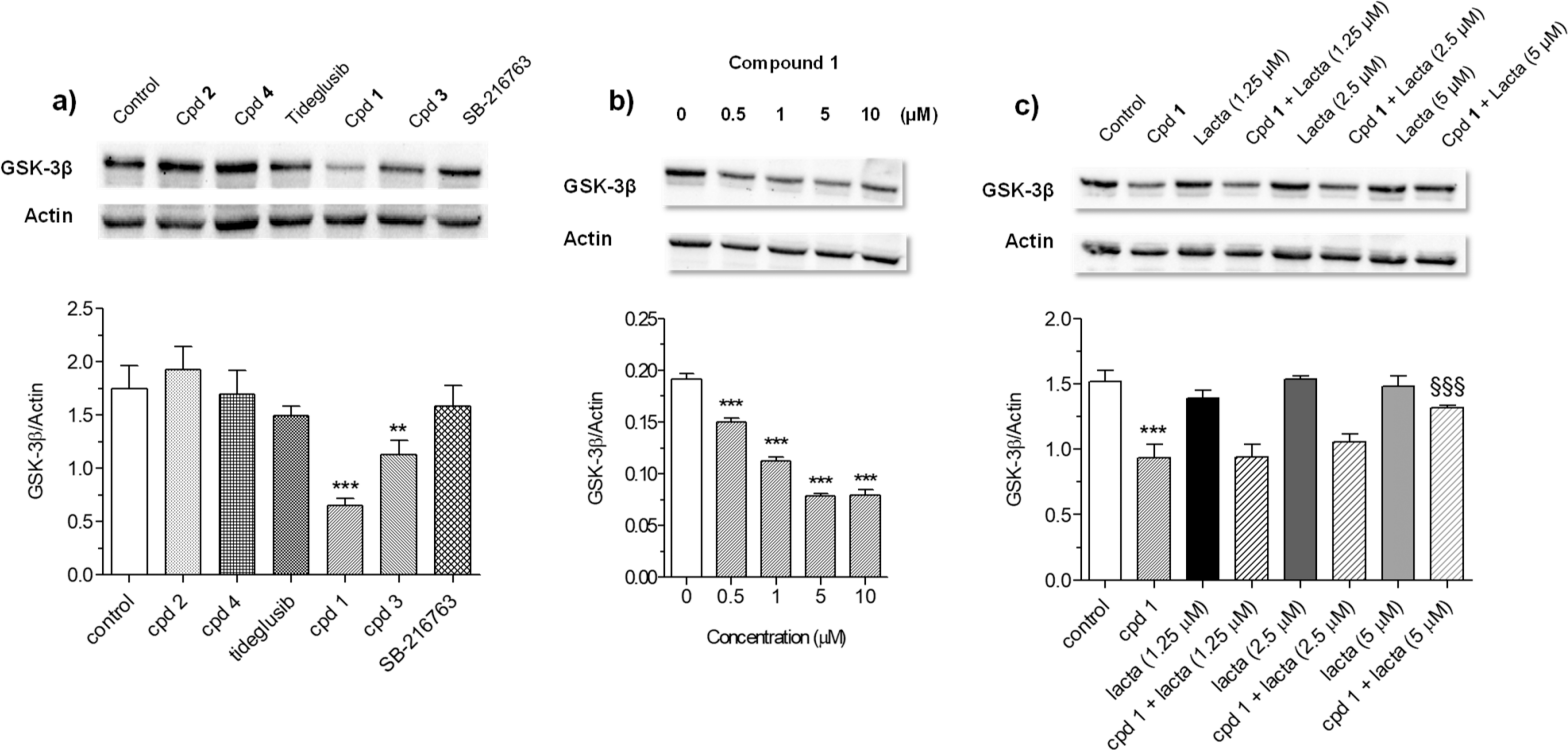
Degradation GSK-3β protein by compounds **1**–**4**, SB-216763, and tideglusib in SH-SY5Y cells. (a) GSK-3β protein level after 48 h of treatment with all compounds (10 μM); (b) GSK-3β protein level after 48 h of treatment with compound **1** (0.5–10 μM); (c) GSK-3β protein level after 24 h of treatment with compound **1** (10 μM) and lactacystin (1.25–5 μM). GSK-3β protein level was measured by Western blotting. Data are expressed as mean ± SEM of three independent experiments (**p < 0.01 and ***p < 0.001 vs untreated cells, ^§§§^*p* < 0.001 vs cells treated with compound **1**, at one-way ANOVA with Dunnett or Bonferroni post hoc test).

**Figure 3:**
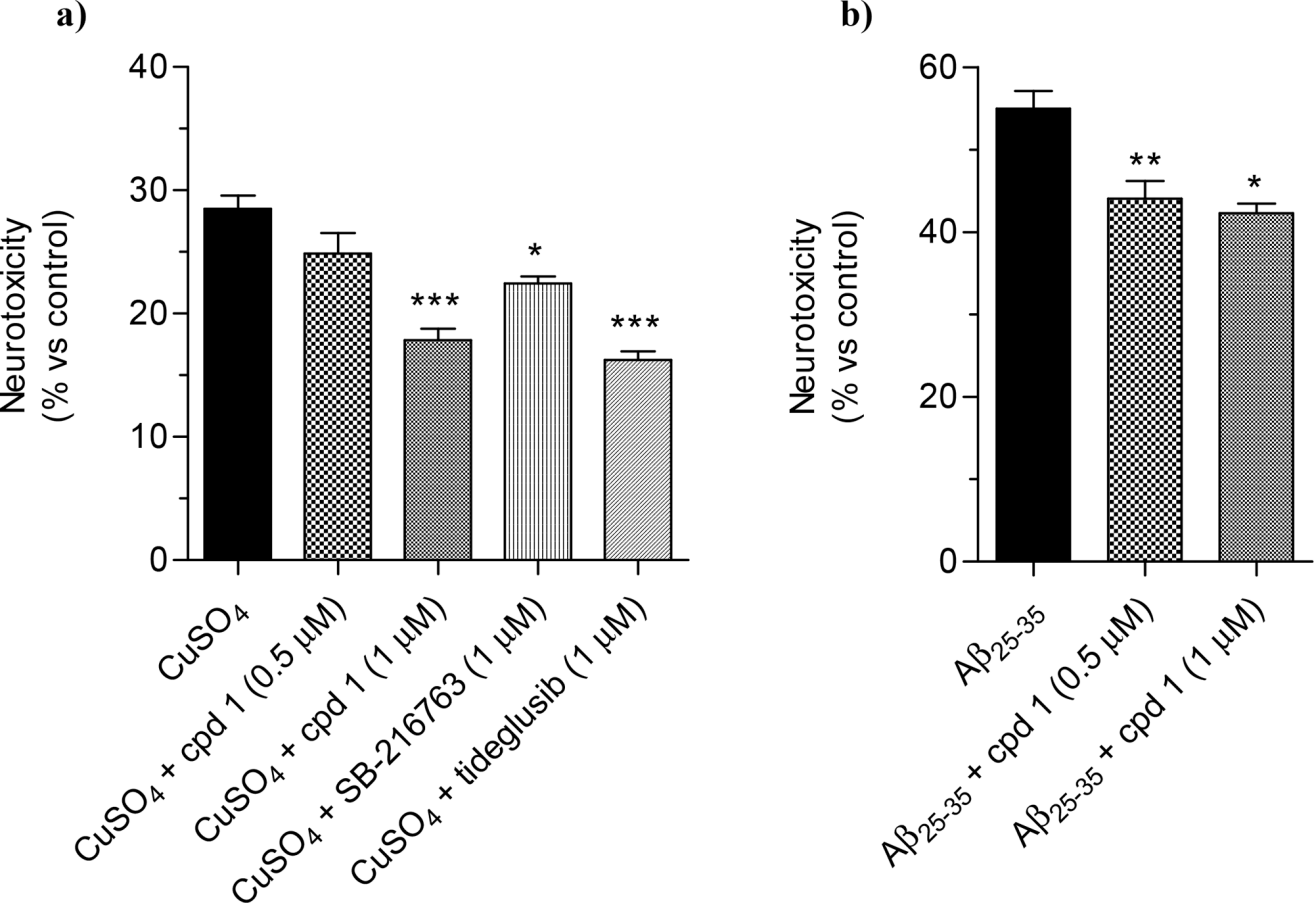
Compound **1** reduced the neurotoxicity induced by CuSO_4_ and Aβ_25–35_ in SH-SY5Y cells. (a) Cells were incubated for 24 h with compound **1** (0.5–1 μM), SB-216763, and tideglusib (1 μM) in the presence of CuSO_4_ (150 μM); (b) cells were incubated for 2 h with compound **1** (0.5–1 μM) and further 3 h in the presence of Aβ_25–35_ (10 μM). At the end of incubation, the cell viability was measured by MTT assay as described in the Methods section. Data are reported as mean ± SEM of three independent experiments (*p < 0.05 and ***p < 0.001 vs cells treated with CuSO_4_; *p < 0.05 and **p < 0.01 vs cells treated with Aβ_25–35_ at one-way ANOVA with Dunnett post hoc test).

First, to have indications about the possible conformations that the designed PROTACs would assume in solution, we submitted **1**–**4** to 200 ns long MD simulations in replicate.^20,21^ Given the presence of several aromatic rings in pomalidomide, SB-216763, and tideglusib, it is likely that the formation of extended stacking interactions leads to bent PROTAC conformations, less able to interact with the corresponding targets. The analysis of intramolecular H-bonds, solvent accessible surface area (SASA; Figure 1–3SI), and radius of gyration (*R*_g_) values (Figure 4SI) pointed out that PROTACs **1** and **2**, featuring a longer linker, might adopt a more open and extended conformation compared to **3** and **4**, carrying a shorter linker. Next, to investigate the orientation of the designed PROTACs at GSK-3β, molecular modeling studies were performed. SB-216763 is known to bind the protein orthosteric site,^17^ and more importantly, the X-ray structures of GSK-3β complexed with maleimide-based inhibitors similar to SB-216763 (PDB codes 1r0e and 1q4l) are available.^22^ Thus, we docked SB-216763-based PROTACs **1** and **3** at GSK-3β, observing that the compound core was able to properly fit the binding site, forming a bidentate H-bond with the Asp133 and Val135 backbone, plus hydrophobic contacts with Ile62, Val70, Lys85, Val110, Leu188, and Cys199. The linker and the pomalidomide moiety can assume different poses, spanning the protein outside surface. The best obtained **1**–GSK-3β and **3**–GSK-3β complexes (in terms of number of formed interactions and docking scores) were then submitted to 200 ns long plain molecular dynamics (MD) simulations to verify and compare the ligand behavior, according to the different linker lengths. As shown in Figure 1b, the SB-216763 core is able to form H-bonds and hydrophobic contacts in the binding site. The linker gains additional contacts with Tyr134 for compound **1** and with Pro136 and Arg141 for **3** (Figure 1b). The results of the *in silico* studies might suggest a higher propensity for compound **1**, because of the longer linker, to orient the pomalidomide moiety toward the kinase β-hairpin loop, interacting with Lys74 and with Gly79 also during MD simulations (Figure 1b, left panel). In contrast, compound **3**’s shorter linker does not allow the pomalidomide moiety to reach the β-hairpin loop, remaining for most of the simulation trapped in a cavity lined by Arg141, His145, and Arg148 (Figure 1b, right panel). Furthermore, the proximity of the pomalidomide moiety to the β-hairpin loop in **1**–GSK-3β complex might suggest **1** to be more likely to bind CRBN, stabilizing a ternary complex. Similarly to the β-hairpin loop of CK-1α reported to bind the carbon terminal domain of CRBN (PDB ID: 5fqd)^23^ leading to CK-1α degradation,^23,24^ we might suppose the β-hairpin loop could play a similar role for GSK-3β, which shares a high 3D structure similarity with CK-1α. Unfortunately, we could not perform the same investigation on tideglusib-derived PROTACs **2** and **4**, as no experimental data or models have unequivocally defined the binding mode of tideglusib with GSK-3β^25^ and no crystal structures have been solved.

### Synthesis

**Scheme 1:**
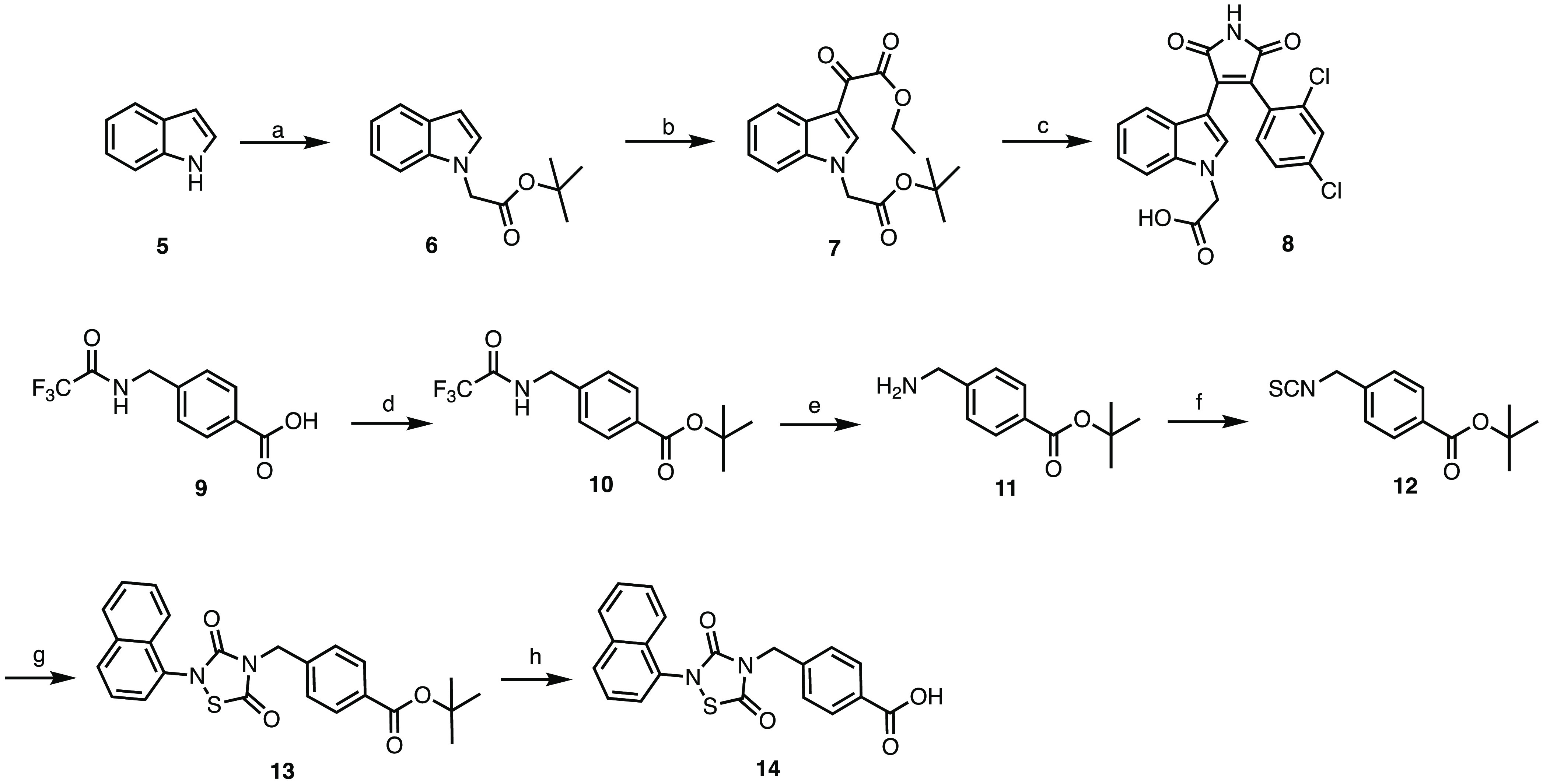
Synthesis of the Precursors **8** and **14** Reagents and conditions. (a) *tert*-butyl bromoacetate, K_2_CO_3_, acetone, reflux, 12 h, 50% yield; (b) ethyl chlorooxoacetate, diethyl ether, rt, 12 h, N_2_, 42% yield; (c) 2-(2,4-dichlorophenyl)acetamide, KOtBu, DMF, rt, 12 h, N_2_, 39% yield; (d) *tert*-butanol, EDCI, DMAP, THF, rt, 12 h, 77% yield; (e) K_2_CO_3_, H_2_O/MeOH, rt, 12 h, 79% yield; (f) 1,1′-thiocarbonyldi-2(1*H*)-pyridone, DCM, rt, 12 h, 55% yield; (g) naphthyl isocyanate, sulfuryl dichloride, THF, rt, 12 h, N_2_ then air, THF, rt, 30 min, 57% yield; (h) TFA, DCM, rt, 12 h, 81% yield.

**Scheme 2:**
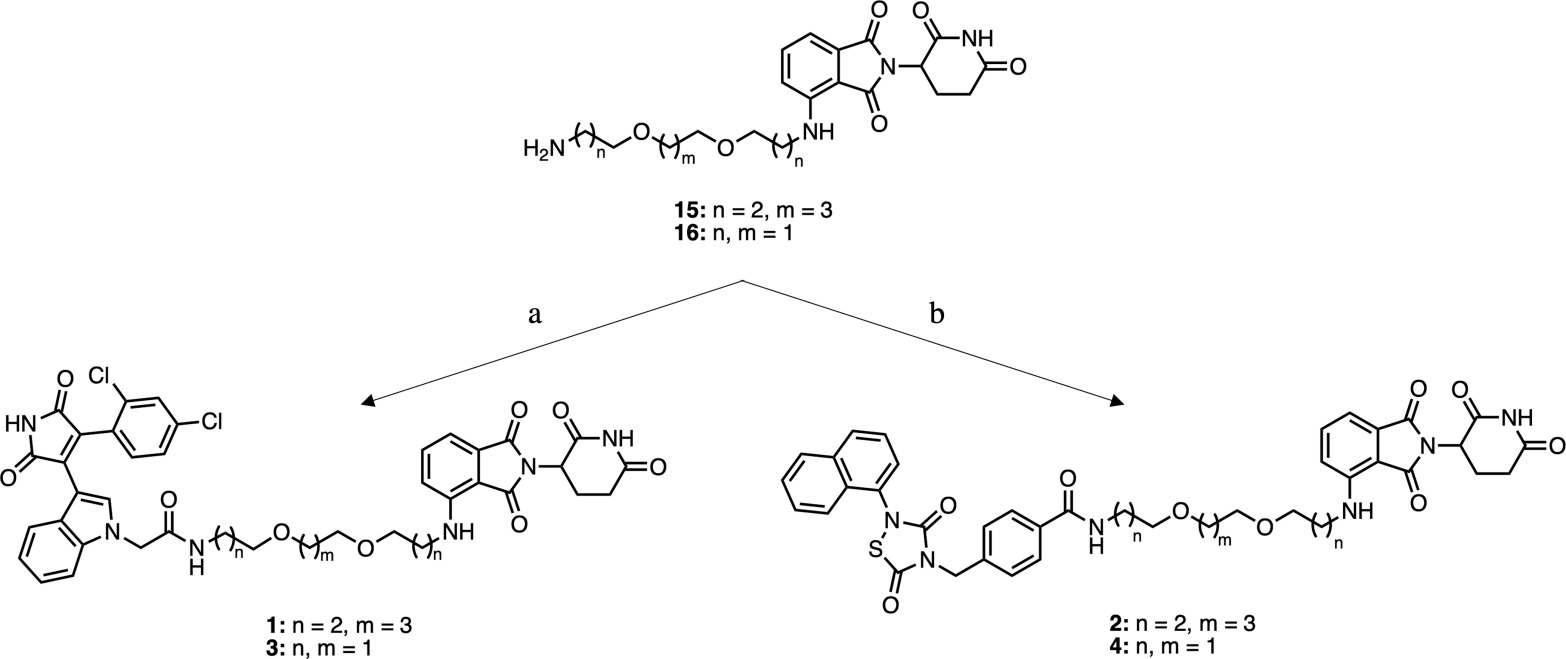
Synthesis of the Target Compounds **1**–**4** Reagents and conditions. (a) **8**, EDCI, HOBT, DIPEA, DMF, rt, 12 h, 32% yield for **1**, 25% yield for **3**; (b) **14**, EDCI, HOBT, DIPEA, DMF, rt, 12 h, 48% yield for **2**, 44% yield for **4**.

Designed compounds **1**–**4** have been synthesized following the sequences reported in Schemes 1 and 2, starting with the synthesis of fragments **8** and **14**. For the synthesis of SB-216763-like fragment **8**, commercially available indole **5** was alkylated with *tert*-butyl bromoacetate in potassium carbonate to give **6** that was then treated with ethyl chlorooxoacetate to give glyoxalate **7**. Perkin-type condensation between **7** and 2-(2,4-dichlorophenyl) acetamide generated the desired deprotected maleimide **8**. To obtain tideglusib-like fragment **14**, we started from trifluoroacetamide derivative **9**(26) that was protected at the carboxylic acidic functionality by treatment with *tert*-butanol to obtain **10**. Basic hydrolysis of the trifluoroacetamide protecting group generated the free primary amino ester **11** that was transformed in isothiocyanate **12** following treatment with 1,1′-thiocarbonyl-di-2(1*H*)-pyridone. The thiadiazolidinone **13** was obtained via a straightforward cyclization/oxidation sequence involving first a cyclization between isothiocyanate **12** and the commercially available naphthyl isocyanate in the presence of sulfuryl chloride and then the oxidation of the obtained product under atmospheric oxygen. The thiadiazolidinone **13** was then deprotected in trifluoroacetic acid to obtain fragment **14** ready to be coupled via linker to the E3 ligase recruitment element.

EDCI/HOBT assisted amide formation between the common intermediates **15**(27) and **16**(27) and the GSK-3β recruiting elements **8** and **14** led to the target compounds **1**–**4** (Scheme 2).

### Biological Evaluation

To check whether the structural modification generated by introducing the ligase recruiting element on both inhibitor scaffolds would lead to impaired target recognition, we evaluated the ability of **1**–**4** and parent inhibitors (SB-216763 and tideglusib) to block GSK-3β activity (Table 1).

**Table 1:**
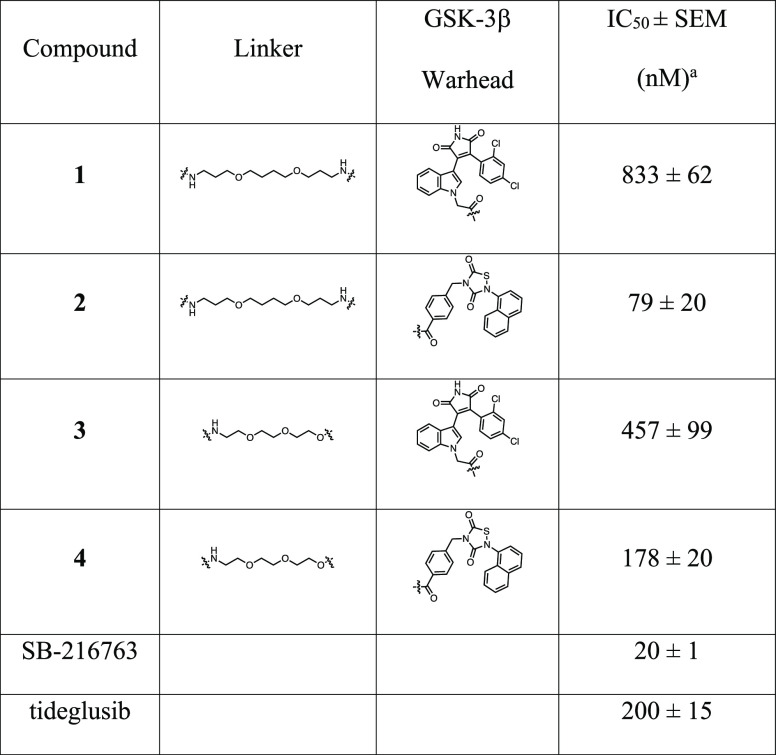
Effects of Compounds **1**–**4**, SB-216763, and Tideglusib on GSK-3β Activity

a IC_50_ value is defined as the drug concentration that reduces by 50% the target activity. The results are reported as the mean value of at least two determinations each carried out in duplicate.

Compounds displayed anti-GSK-3β activity spanning from micromolar to nanomolar scale. Activity was clearly influenced by GSK-3β warhead and linkers. The structural modification introduced on the maleimide scaffold is highly detrimental for GSK-3β inhibitory activity: the PROTACs **1** and **3** are characterized by IC_50_ values (**1**: 833 nM; **3**: 457 nM) which are <1 order of magnitude higher than the starting inhibitor SB-21676 (20 nM). Interestingly, the length of the linker has an impact on the interaction with GSK-3β, since compound **1**, characterized by the 3–4–3 PEG linker, is less active than compound **3**, characterized by the shorter 2–2–2 linker. On the other hand, the introduction of the linker–E3 ligase recruiting moiety on tideglusib significantly increases the inhibitory activity in the case of 3–4–3 PROTAC (**2**), when tested in the same assay conditions (**2**: 79.26 nM; tideglusib: 200 nM). This does not apply to 2–2–2 PROTAC **4**, where the structural modification has no significant impact on the inhibitory activity (IC_50_**4**: 177.56 nM). Overall, the inhibitory profiles displayed by the four PROTACs seem sufficient to engage GSK-3β and allow the formation of the ternary GSK-3β–PROTAC–E3 ligase complex, which is necessary to trigger the degradation process. Indeed, one of the advantages of PROTACs over classical inhibition is that, while traditional small molecule inhibitors need to form strong interactions with their biological counterparts, PROTACs may only require moderate binding to the POI to catalytically induce its degradation.^28^

Considering that (neuro)toxicity of AD drug candidates has been a drawback for clinical translation, cytotoxicity of compounds **1**–**4**, SB-216763, and tideglusib was evaluated in neuronal SH-SY5Y cells (Figure 5SI). Viability was measured using the MTT (3-(4,5-dimethylthiazol-2-yl)-2,5-diphenyl-tetrazolium bromide) reduction assay after 24 h of treatment with various concentrations of compounds **1**–**4**, SB-216763 and tideglusib (1.25–40 μM). Compounds **1**, **3**, **4**, and SB-216763 showed cytotoxicity at concentrations higher than 20 μM, while for compound **2** cytotoxicity was detected already at 20 and 40 μM. Before performing the degradation assay, we confirmed that 48 h treatment of SH-SY5Y cells with **1**–**4** at 10 μM concentration did not significantly modify cell viability (data not shown). Then, the ability of compounds **1**–**4**, SB-216763, and tideglusib to induce the degradation of GSK-3β was evaluated in terms of total GSK-3β protein level decrease by Western blotting (Figure 2a). Inhibitors SB-216763 and tideglusib did not modify the basal level of GSK-3β protein. Among the PROTACs, only **1** and **3** (and not **2** and **4**) significantly decreased the total level of GSK-3β protein, suggesting the specific ability of the two SB-216763-derived compounds to degrade GSK-3β protein. Compound **1** showed a higher activity in comparison to **3**, maybe due to the formation of a more stable GSK-3β–**1**–E3 ligase complex (see Computational Analysis). There are many potential reasons why tideglusib-derived **2** and **4** did not induce GSK-3β degradation. The one related to their binding to an allosteric site should be ruled out, since PROTAC efficacy depends on POI recruitment, irrespective of the binding site. In fact, there are several positive examples of allosteric PROTACs, such as the allosteric EGFR degrader reported by Gray et al. in 2020.^29^ A plausible explanation could rely on the fact that the selected linkers are not the suitable ones, considering the critical role the linker plays on the formation of the ternary complex. Furthermore, the availability (lacking for tideglusib) of an X-ray structure that characterizes the binding mode of the recruiting element in complex with its target is a fundamental prerequisite for PROTAC design and assembly. As a general remark, these data support the view that the selection of a suitable kinase inhibitor, as well as ample structural variations on the linkers, including length, flexibility, and attachment points, are crucial for the development of effective degraders.^30^ Clearly, a means to answer these questions is to experimentally assess ternary complex formation.^31^

We further investigated the dose-dependent GSK-3β degradation capacity of **1**, by treating SH-SY5Y cells for 48 h at 0.5, 1, 5, and 10 μM concentrations. As shown in the Western blot of Figure 2b, compound **1** significantly decreased GSK-3β protein level at all tested concentrations in a dose-dependent manner, and with a half-maximal degradation (DC_50_) of 6.22 μM. This seems to support a consistent degradation effectiveness.

To confirm the involvement of the UPS in the GSK-3β degradation, SH-SY5Y cells were treated for 24 h with compound **1** (10 μM) in the presence of increased concentration of lactacystin (1.25–2.5–5 μM), a potent and selective irreversible 20S proteasome inhibitor. As shown in Figure 2c, the treatment with compound **1** in the presence of lactacystin at a concentration of 5 μM had no effect on the total GSK-3β protein level, indicating that the GSK-3β degradation induced by compound **1** involves the UPS. Interestingly, being that GSK-3β degradation induced by **1** is directly proportional to the dose of lactacystin, the involvement of the UPS in the mechanism of degradation seems confirmed.

Several studies have reported that GSK-3β is involved in tau protein phosphorylation and neuronal death in AD.^3,4^ In this regard, *in vitro* and *in vivo* studies have demonstrated the role of copper to exacerbate tau hyperphosphorylation, ultimately contributing to both synaptic failure and neuronal death.^32^ Based on this evidence, to assess the effect of compound **1** on neuronal death induced by copper, SH-SY5Y cells were incubated for 24 h with compound **1** (0.5–1 μM), SB-216763, and tideglusib (1 μM) in the presence of copper sulfate (CuSO_4_, 150 μM), and cell viability was measured by MTT assay.^14^ As shown in Figure 3a, compound **1**, SB-216763, and tideglusib significantly counteracted the neurotoxicity induced by CuSO_4_ at 1 μM. Although we did not check whether copper sulfate induced GSK-3β upregulation, it is encouraging that, remarkably, the neuroprotective effect of **1** was abolished by lactacystin (5 μM) (Figure 6SI). In parallel, the neuroprotective activity of compound **1** was also evaluated against insult from Aβ_25–35_ peptide, the neurotoxic fragment of Aβ involved in the neuropathology of AD. GSK-3β is aberrantly activated by the presence of Aβ and contributes to neural damage.^33^ Thus, SH-SY5Y cells were incubated for 2 h with compound **1** (0.5 and 1 μM) and further 3 h with Aβ_25–35_ peptide (10 μM). At the end of incubation, cell viability was evaluated by MTT assay. The treatment with both concentrations of **1** markedly reduced the neurotoxicity induced by Aβ_25–35_ peptide, in a dose-dependent manner (Figure 3b).

For AD-directed drugs, the ability to cross the blood–brain barrier (BBB) is a fundamental prerequisite. We tested SB-216763-derived PROTACs **1** and **3** in BBB specific parallel artificial membrane permeability assay (PAMPA-BBB) (Supporting Information Table 1 and Figure 7SI). Compound **1** had an effective permeability (*P*_e_) of 15.33 ± 1.12, while compound **3** had a *P*_e_ of 20.68 ± 3.93. Based on these results, we can classify compound **1** as CNS± permeable approaching CNS+ permeability values (Figure 8SI), while **3** is predicted to cross the BBB.

## Conclusions

GSK-3β has become one of the most investigated AD targets by both companies and academia, due to its critical roles in cellular homeostasis and in a multitude of neurodegeneration-specific signaling pathways. Despite this, up to now, no GSK-3β inhibitor has been approved for clinical practice. In recent years, the PROTAC paradigm has emerged as a compelling strategy for modulating challenging or traditionally considered “undruggable” targets. Based on this approach, a POI is degraded rather than being simply blocked, leading to a number of advantages over classical inhibition. Following the explosion of the PROTAC paradigm,^34^ we applied this strategy to the development of GSK-3β-directed degraders based on the structure of two chemically and mechanistically different GSK-3β inhibitors, i.e., SB-216763 and tideglusib. The obtained compounds **1**–**4**, which employ pomalidomide as CRBN E3 ligase targeting element, show a good level of POI engagement (as indirectly evaluated via enzymatic assay). Compound **1**, characterized by the SB-216763 recruiting moiety and the 3–4–3 PEG linker, is not toxic and is the most potent degrader of the set, able to induce significant GSK-3β degradation already at 0.5 μM and in a dose-dependent manner. Moreover, by using a specific proteasome inhibitor, we demonstrated that GSK-3β degradation is mediated by the UPS. Finally, PROTAC **1** is effective in two disease cell models: in SH-SY5Y cells, it is able to counteract toxic insults induced by Cu^2+^ and Aβ_25–35_ at the low concentrations of 1 and 0.5 μM, respectively.

Above all, these results demonstrated that SB-216763-based PROTACs are potent GSK-3β degraders, and further SAR optimization, together with development of PK–PD relationships, is underway to obtain GSK-3β degraders for preclinical development.

## Methods

Procedures for the synthesis of targets compounds **1**–**4** and their characterization, *in vitro* and *in cell* assays, PAMPA-BBB assay, and computational studies are included in the Supporting Information.

## Supporting Information Available

Experimental procedures for chemistry, biology and computational analysis, compounds synthesis and characterization, NMR spectra, and supplementary figures (PDF)

## Abbreviations

Aβ: amyloid β
AD: Alzheimer’s disease
CK-1α: casein kinase 1α
CRBN: cereblon
GSK-3β: glycogen synthase kinase 3β
MD: molecular dynamics
MTT: (3-(4,5-dimethylthiazol-2-yl)-2,5-diphenyl-tetrazolium bromide
PROTAC: proteolysis-targeting chimera
POI: protein of interest
UPS: ubiquitin–proteasome system
