# Supplementary material for: PROTAC-Induced Glycogen Synthase Kinase 3β Degradation as a Potential Therapeutic Strategy for Alzheimer’s Disease
Source: ACS Chem Neurosci. 2023 May 23;14(11):1963–70. doi: 10.1021/acschemneuro.3c00096 (PMC10251479; doi:10.1021/acschemneuro.3c00096)
Supplement: Supplementary file 1 — cn3c00096_si_001.pdf [file cn3c00096_si_001.pdf]

PROTAC-INDUCED GLYCOGEN SYNTHASE KINASE 3β DEGRADATION AS A POTENTIAL THERAPEUTIC STRATEGY FOR ALZHEIMER'S DISEASE

Melissa Guardigni,[1‡] Letizia Pruccoli,[1‡] Alan Santini,[1] Angela De Simone,[2] Matteo Bersani,[2] Francesca Spyrakis,[2] Flavia Frabetti,[3] Elisa Uliassi,[4] Vincenza Andrisano,[1] Barbara Pagliarani,[1] Paula Fernández-Gómez,[5] Valle Palomo,[5, 6] Maria Laura Bolognesi,[4*] Andrea Tarozzi,[1] Andrea Milelli[1*]

1 Department for Life Quality Studies, *Alma Mater Studiorum-University of Bologna*, Corso d'Augusto 237, 47921 Rimini

2 Department of Drug Science and Technology, *University of Turin*, Via Pietro Giuria 9, 10125 Torino

3 Department of Medical and Surgical Sciences, *Alma Mater Studiorum-University of Bologna*, Via Belmeloro 8, 40126 Bologna

4 Department of Pharmacy and Biotechnology, *Alma Mater Studiorum-University of Bologna*, Via Belmeloro 6, 40126, Bologna

5 Instituto Madrileño de Estudios Avanzados en Nanociencia (IMDEA-Nanociencia), C/Faraday 9, 28049 Madrid, Spain.Madrid, Spain.

6 Centro de Investigación Biomédica en Red de Enfermedades Neurodegenerativas (CIBERNED), Instituto de Salud Carlos III, Av. de Monforte de Lemos, 5, 28029, Madrid, Spain, Madrid, Spain

Table of contents

1. Figure 1SI. hydrophilic SASA values calculated for compounds **1**-**4**.

2. Figure 2SI. Number of intramolecular H-bonds and donor-acceptor pairs determined for compounds **1**-**4**.

3. Figure 3SI. Map of the intramolecular H-bonds (IHBs) of compounds **1-4** identified from 200ns MD simulation with occupancy higher than 0.5%.

4. Figure 4SI. Radius of gyration (grey lines) determined for compounds **1**-**4**.

5. Figure 5SI. Cytotoxicity of compounds **1-4**, SB-216763 and tideglusib in SH-SY5Y cells.

6. Figure 6SI. Effect of Lactacystin on the neuroprotective effects of compound **1**

7. Chemistry.

8. Copies of ^{1}H-NMR and ^{13}C-NMR spectra.

9. Computational analysis.

10. Biology.

11. PAMPA BBB permeation studies.

12. References

1.

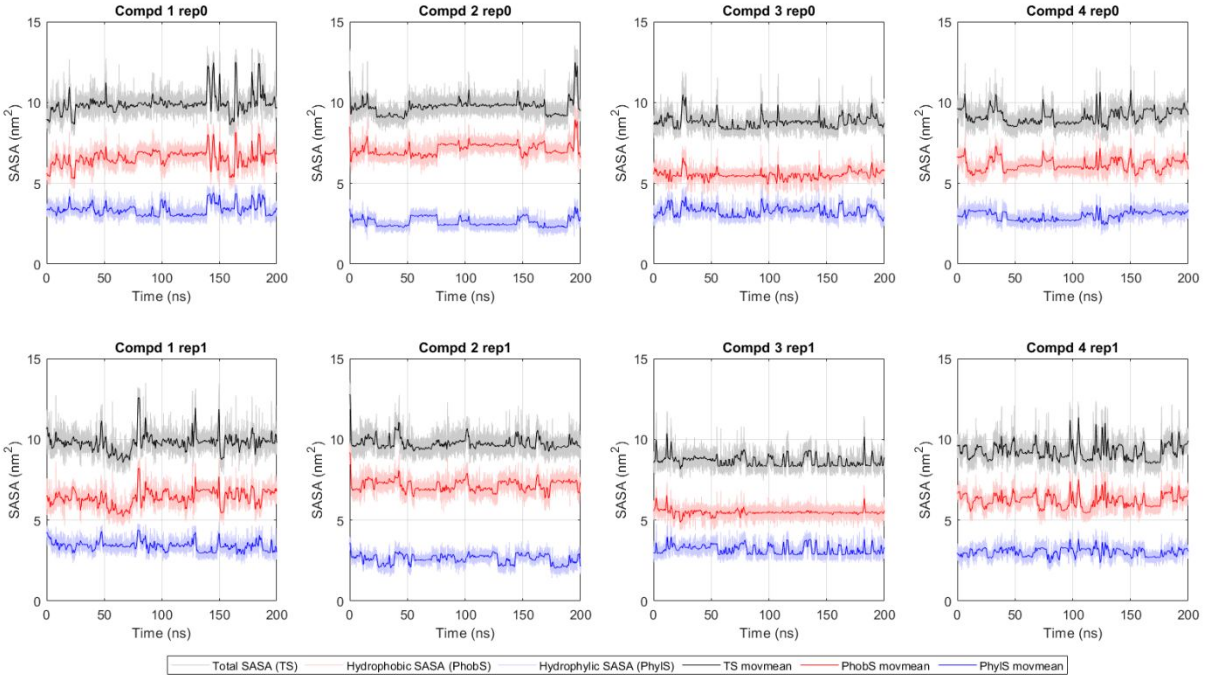

Figure 1SI. Total, hydrophobic and hydrophilic SASA values (grey, light red and light blue lines, respectively) calculated for compounds **1**-**4** along the MD trajectories. The corresponding moving means are represented black, red and blues lines.

2.

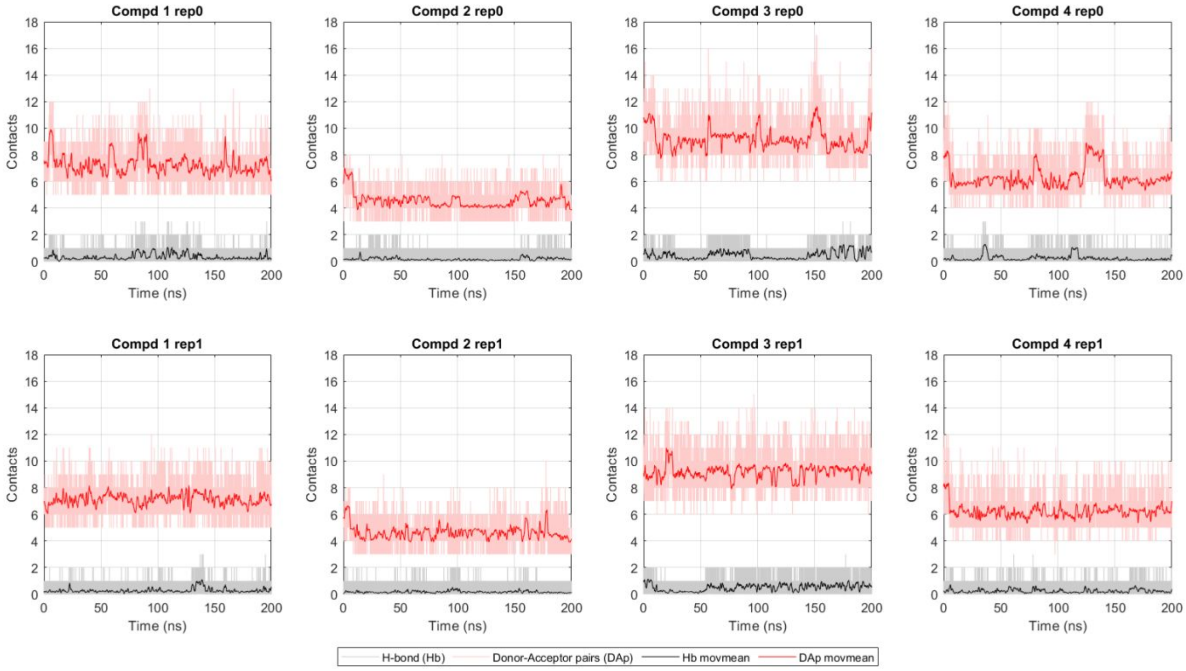

Figure 2SI. Intramolecular H-bonds satisfying both distance and angle criteria (Hb, grey lines), and donor-acceptor pairs (Dap; light red lines) within 0.35 nm determined for compounds **1-4**, along the MD trajectories. The corresponding moving means are represented by black and red lines, respectively.

3.

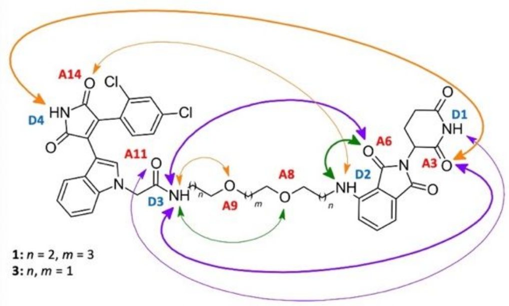

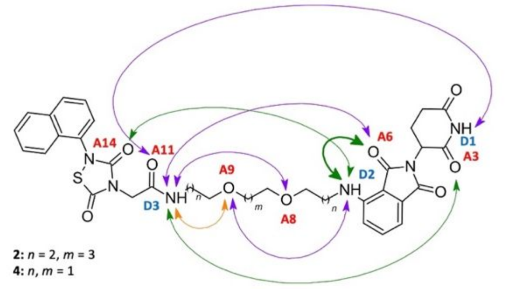

SB-series	A3	A6	A8	A9	A11	A14
D1					3 rep0 1.6	
D2		1 rep0 16.8<br>1 rep1 17.6<br>3 rep0 18.8<br>3 rep1 18.3				1 rep0 0.6<br>1 rep1 0.5
D3	3 rep0 2.8<br>3 rep1 8.4	3 rep0 13.8<br>3 rep1 26.3	1 rep0 0.7<br>3 rep0 1.5<br>3 rep1 1.0	1 rep0 2.9<br>1 rep1 1.5		
D4	1 rep0 11.3<br>1 rep1 5.4					

Tideg-series	A3	A6	A8	A9	A11	A14
D1					4 rep0 0.5	
D2		2 rep0 11.2<br>2 rep1 11.7<br>4 rep0 10.6<br>4 rep1 11.4		4 rep0 1.2<br>4 rep1 1.0		2 rep0 1.1<br>2 rep1 0.5<br>4 rep0 1.7<br>4 rep1 0.6
D3	2 rep1 1.0<br>4 rep0 2.6<br>4 rep1 3.1	4 rep0 1.6<br>4 rep1 2.9	4 rep0 4.7<br>4 rep1 3.0	2 rep0 3.6<br>2 rep1 2.4		

Figure 3SI. Map of intramolecular H-bonds (IHBs) of compounds **1-4** identified from 200ns MD simulation with occupancy higher than 0.5%. IHBs of longer linker and shorter linker compounds are reported respectively in orange and violet. IHBs are shown in green if they are detected independently from the linker length. Details regarding occupancy percentages and the replica number are reported in the table. IHBs with average occupancy percentages between replicas higher than 5% are shown using bold arrows (scheme) and are highlighted in darker colors (table).

4.

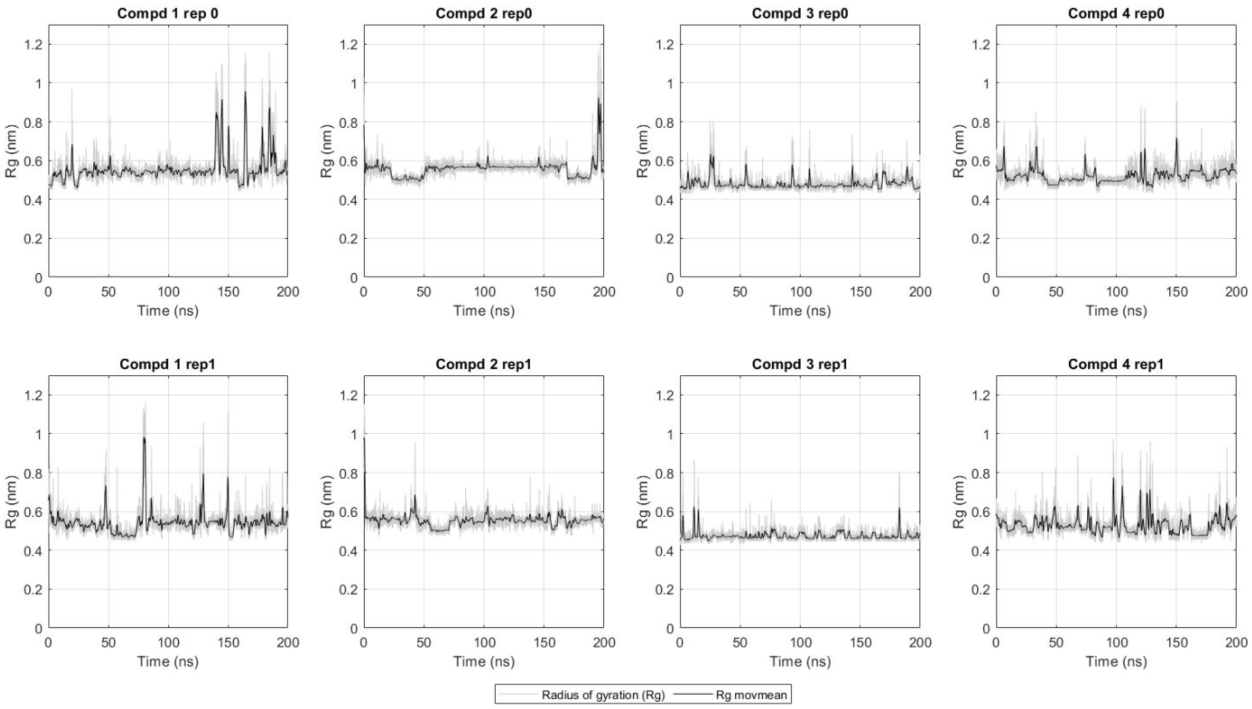

Figure 4SI. Radius of gyration (grey lines) determined for compounds **1**-**4**, along the MD trajectories. The corresponding moving means are represented by black lines.

5.

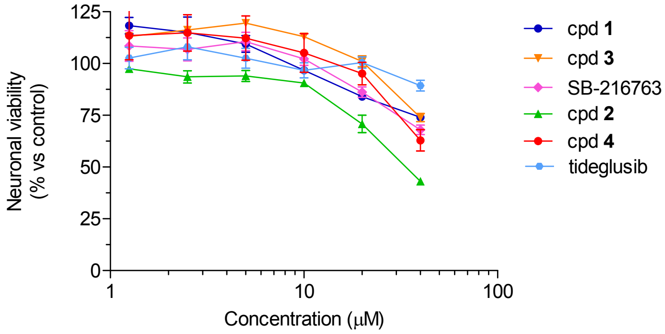

Figure 5SI. Cytotoxicity of compounds **1-4**, SB-216763 and tideglusib in SH-SY5Y cells. Cells were incubated for 24 h with different concentrations of the studied compounds (1.25-40 μM). At the end of incubation, the cell viability was measured by MTT assay as described in the materials and methods section. Data are reported as mean ± SEM of three independent experiments.

6.

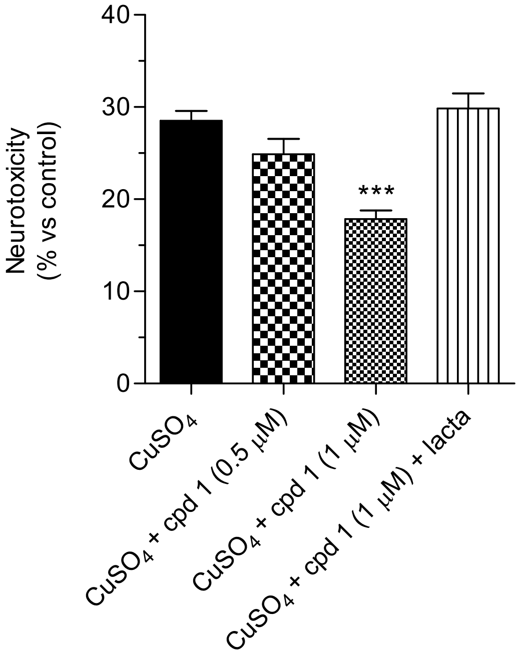

Figure 6SI Lactacystin abolishes the neuroprotective effects of compound **1** against the cytotoxicity induced by CuSO4 in SH-SY5Y cells. Cells were incubated for 24 h with compound **1** (0.5-1 μM) and CuSO4 (150 μM) in presence of lactacystin (lacta, 10 μM). At the end of incubation, the cell viability was measured by MTT assay as described in the materials and methods section. Data are

reported as mean ± SEM of three independent experiments (***p<0.001 vs cells treated with CuSO4; one-way ANOVA with Dunnett post-hoc test).

7. Chemistry

All of the commercially available reagents and solvents were purchased from Sigma-Aldrich, Alpha Aesar, and VWR, and used without further purification. Reactions were followed by analytical thin-layer chromatography (TLC), on precoated TLC plates (layer 0.20 mm silica gel 60 with a fluorescent indicator UV254, from SigmaAldrich). Developed plates were air-dried and analyzed under a UV lamp (UV 254/365 nm). Nuclear magnetic resonance (NMR) experiments were run on a Varian VXR 400 (400 MHz for ^{1}H, 100 MHz for ^{13}C). ^{1}H and ^{13}C NMR spectra were acquired at 300 K using deuterated dimethyl sulfoxide (DMSO-d6), chloroform ($CDCl_3$). Chemical shifts (δ) are reported in parts per million (ppm) relative to tetramethylsilane (TMS) as the internal reference, and coupling constants (J) are reported in hertz (Hz). The spin multiplicities are reported as s (singlet), br s (broad singlet), d (doublet), t (triplet), q (quartet), and m (multiplet). Low-resolution and high resolution mass spectra were recorded on a VG707EH-F or a Xevo G2-XS QTof apparatus, and electrospray ionization (ESI) both in positive and negative modes was applied. All the final compounds showed ≥95% purity by analytical HPLC. Compounds were named following IUPAC rules as applied by ChemBioDraw Ultra (version 19.0).

***tert*-butyl 4-((2,2,2-trifluoroacetamido)methyl)benzoate (10).** To a solution of **9** (1 g, 4.0 mmol) in anhydrous THF under stream of nitrogen, DMAP (0.49 g, 4.0 mmol) and tert-Butanol (10 mL) were added at 0 °C. Shortly after EDCI (2.31 g, 12.0 mmol) and the reaction was allowed to reach rt and stirred overnight. Solvents were removed in vacuo and the resulting residue was taken up with DCM and washed with HCl_{aq} 1N, water and brines. Organic phase was dried over Na_2SO_4 and concentrated to give **10** (0.960 g, 77% yield) as yellow oil. ^{1}H-NMR (400 MHz, $CDCl_3$) δ 1.57 (s, 9H), 4.54 (d, 2H, J = 6.4 Hz), 7.28-7.31 (dd, 2H, J = 8.0, 0.4 Hz), 7.91-7.94 (dd, 2H, J = 8.4, 2.0 Hz);

^{13}C-NMR (100 MHz, $CDCl_3$) d 28.1, 43.4, 81.4, 111.6-121-2 (q, J = 285.6, 276.9 Hz), 127.6, 129.9, 130.0, 140.7, 157.0-158.2 (q, J = 37.4, 37.4 Hz), 165.5; ESI-MS = 304 $(M+H)^+$.

***tert*-butyl 4-(aminomethyl)benzoate (11).** To a solution of **10** (0.885 g, 2.9 mmol) in a mixture of methanol / water (30 ml, 17:1 ratio), potassium carbonate (1.61 g, 12 mmol) was added at 0 °C and the reaction was allowed to reach rt and stirred overnight. Methanol was removed in vacuo and the aqueous phase was extract with DCM. Organic phases were collected, dried over Na_2SO_4 and concentrated to give **11** (0.480 g, 79% yield) as yellow oil. ^1H-NMR (400 MHz, $CDCl_3$) δ 1.58 (s, 9H), 3.95 (s, 2H), 7.33 (d, 2H, J = 8.4 Hz), 7.88 (d, 2H, J = 8.4 Hz); ^{13}C-NMR (100 MHz, $CDCl_3$) d 28.1, 43.6, 81.1, 127.0, 127.6, 129.8, 131.4, 165.4; ESI-MS = 208 $(M+H)^+$.

***tert*-butyl 4-(isothiocyanatomethyl)benzoate (12).** To a solution of **11** (0.480 g, 2.32 mmol) in DCM under stream of nitrogen, 1,1′-Thiocarbonyldi-2(1*H*)-pyridone (0.533 g, 2.32 mmol) in DCM was added dropwise and the resulting mixture was stirred overnight at rt. The solvent was removed in vacuo and the resulting residue was purified via flash chromatography using as mobile phase a mixture of petroleum ether / ethyl acetate (9.8:0.2) to give **12** (0.320, 55% yield) as yellow oil. ^1H-NMR (400 MHz, $CDCl_3$) δ 1.59 (s, 9H), 4.77 (s, 2H), 7.36 (d, 2H, J = 8.0 Hz), 8.01 (d, 2H, J = 8.0 Hz); ^{13}C-NMR (100 MHz, $CDCl_3$) d 28.1, 48.4, 81.3, 126.5, 130.1, 132.1, 138.5, 165.1; ESI-MS = 250 $(M+H)^+$.

***tert*-butyl 4-((2-(naphthalen-1-yl)-3,5-dioxo-1,2,4-thiadiazolidin-4-yl)methyl)benzoate (13).** A stirred solution of **12** (0.5 g, 2 mmol) and 1-naphthyl isocyanate (0.34 mL, 0.372 g, 2 mmol) under nitrogen in THF was cooled to 0 °C. Sulfuryl chloride in 1M solution of DCM (4 mL) was added slowly and the mixture was allowed to warm to rt and stirred overnight. The reaction was then opened to the air and stirred for 30 minutes before the solvent was removed under vacuo and the obtained residue was purified via flash chromatography using as mobile phase a mixture of petroleum ether / ethyl acetate (9.5:0.5 to 9:1) to give **13** (0.5 g, 57% yield) as yellow oil. ^1H-NMR (400 MHz, $CDCl_3$) δ 1.55 (s, 9H), 4.97 (s, 2H), 7.53 (d, J = 8.4 Hz, 2H), 7.69–7.61 (m, 3H), 7.80 (dd, J = 7.4, 1.2 Hz, 1H), 7.97–7.90 (m, 3H), 8.09 (t, J = 8.8 Hz, 2H); ESI-MS = 436 $(M+H)^+$.

4-((2-(naphthalen-1-yl)-3,5-dioxo-1,2,4-thiadiazolidin-4-yl)methyl)benzoic acid (14). In a stirred solution of **13** (0.50 g, 1.15 mmol) in anhydrous DCM at 0 °C under stream of nitrogen, trifluoroacetic acid was added (2 mL). The reaction was allowed to reach rt and stirred overnight. The reaction mixture was washed with water and the organic phase was dried over Na_2SO_4 and concentrated to give **14** (0.459 g, 81% yield) as yellow oil. ^{1}H-NMR (400 MHz, $CDCl_3$) δ 4.97 (s, 2H), 7.53 (d, 2H, *J* = 8.8 Hz), 7.61-7.67 (m, 3H), 7-80-7.82 (m, 1H), 7.92-7.94 (m, 1H), 7.98 (d, 2H, *J* = 6.4 Hz), 8.07-8.11 (m, 2H); ESI-MS = 377 $(M-H)^-$.

tert-butyl 2-(1H-indol-1-yl)acetate (6). To a solution of indole **5** (2.00 g, 17.00 mmol) in acetone, potassium carbonate (4.71 g, 34.18 mmol) and *tert*-Butyl bromoacetate (5.00 g, 25.6 mmol) were added and the reaction was refluxed for 12 h. The solvent was removed under vacuo and the residue was taken up with ethyl acetate and washed with sodium bicarbonate, brine. The organic phase was dried over Na_2SO_4 and concentrated to give a residue that was purified via flash chromatography using as mobile phase a mixture of petroleum ether / ethyl acetate (9.5:0.5) to give **6** (1.95 g, 50% yield) as yellow oil. ^{1}H-NMR (400 MHz, DMSO) δ 1.42 (s, 9H), 4.99 (s, 2H), 6.43 (dd, 1H, *J* = 3.2, 0.8 Hz), 7.00-7.05 (m, 1H), 7.10-7.14 (m, 1H), 7.30-7.34 (m, 2H), 7.53-7.55 (m, 1H); ^{13}C-NMR (100 MHz, $CDCl_3$) d 28.1, 48.1, 81.8, 101.4, 110.1, 119.6, 120.7, 121.6, 128.5, 130.1, 136.8, 168.5; ESI-MS = 232 $(M+H)^+$.

ethyl 2-(1-(2-(*tert*-butoxy)-2-oxoethyl)-1*H*-indol-3-yl)-2-oxoacetate (7). To a solution of **6** (1.00 g, 4.33 mmol) in diethyl ether at 0 °C and under a stream of nitrogen, ethyl chlorooxoacetate (2.40 mL, 2.94 g, 21.65 mmol) was added over 15 minutes and the reaction was allowed to reach rt and stirred for 2 h. The reaction was washed with water and dried to give a residue that was purified via flash chromatography using as mobile phase a mixture of petroleum ether / ethyl acetate (9:1 to 85:15) to give **7** (0.60 g, 42% yield). ^{1}H-NMR (400 MHz, $CDCl_3$) δ 1.42 (s, 9H), 1.51 (t, 3H, *J* = 7.1 Hz), 4.31 (q, 2H, J = 7.0 Hz), 5.03 (s, 2H), 6.47 (dd, 1H, *J* = 3.2, 0.8 Hz), 6.99-7.08 (m, 1H), 7.11-7.16 (m, 1H), 7.32-7.38 (m, 2H), 7.50-7.57 (m, 1H).

2-(3-(4-(2,4-dichlorophenyl)-2,5-dioxopyrrolidin-3-yl)-1*H*-indol-1-yl)acetic acid (8). To a solution of **7** (0.535 g, 1.60 mmol) and 2-(2,4-dichlorophenyl)acetamide (0.363 g, 1.78 mmol) in THF at 0 °C and under a stream of nitrogen, potassium tert-butoxide (0.448 g, 4.0 mmol) in DMF was added and the mixture was stirred at rt for 4 h. HCl 1N was added and the mixture was extracted with ethyl acetate and washed with brine. The organic phase was dried over Na_2SO_4 and concentrated to give **8** as free acid (0.257 g, 39%). ^{1}H-NMR (400 MHz, $CDCl_3$) δ 4.75 (s, 2H), 6.29 (d, 1H, *J* = 8.0 Hz), 6.71 (t, 1H, *J* = 7.6 Hz), 7.04 (t, 1H, *J* = 7.2 Hz), 7.28-7.50 (m, 3H), 7.70 (d, 1H, *J* = 2.4 Hz), 8.10 (s, 1H).

General procedure for the synthesis of 1-4. To the of the corresponding acids **8**, **14** and amines **15**, **16** in DMF, DIPEA, EDCI and HOBT were added a at 0 °C and under a stream of nitrogen. Then the mixture was allowed to warm to rt and stirred overnight. Ethyl acetate and water were added, and the organic phase was washed with brine, dried over Na_2SO_4 and concentrated to give a residue that was purified via flash chromatography using as mobile phase a mixture of petroleum ether / ethyl acetate to give the corresponding PROTACs **1-4** as yellow oil.

2-(3-(4-(2,4-dichlorophenyl)-2,5-dioxo-2,5-dihydro-1*H*-pyrrol-3-yl)-1*H*-indol-1-yl)-*N*-(3-(4-(3-((2-(2,6-dioxopiperidin-3-yl)-1,3-dioxoisoindolin-4-yl)amino)propoxy)butoxy)propyl)acetamide (1). Synthesized from **8** (0.095 g, 0.23 mmol), **15** (0.155 g, 0.23 mmol), DIPEA (0.12 mL, 0.089 g, 0.69 mmol), EDCI (0.087 g, 0.46 mmol), HOBT (0.031 g, 0.23 mmol). Obtained 0.074 g of yellow oil (32% yield). ^{1}H-NMR (400 MHz, DMSO) δ 1.51-1.54 (m, 4H), 1.62-1.66 (m, 2H), 1.78-1.81 (m, 2H), 2.00-2.03 (m, 1H), 3.11-3.14 (m, 2H), 3.29-3.35 (m, 11H), 3.42-3.45 (m, 1H), 4.94 (s, 2H), 5.02-5.06 (dd, 1H, J = 12.8, 5.6 Hz), 6.36-6.38 (d, 1H, J = 8.0 Hz), 6.65 (t, 1H, J = 6.0 Hz), 6.77 (t, 1H, J = 7.6 Hz), 7.00-7.02 (d, 1H, J = 6.8 Hz), 7.06-7.10 (m, 2H), 7.35-7.41 (m, 2H), 7.46-7.48 (dd, 1H, J = 8.4, 2.0 Hz), 7-52-7.58 (m, 2H), 7.72-7.73 (d, 1H, J = 2.0 Hz), 8.12 (s, 1H), 8.27 (t, 1H, J = 5.6 Hz), 11.08 (s, 1H), 11.19 (s, 2H); ^{13}C-NMR (100 MHz, DMSO) δ 22.15, 25.97, 26.04, 28.88, 29.21, 30.98, 36.09, 48.53, 49.04, 67.46, 67.86, 69.87, 70.03, 104.38, 109.08, 110.38, 110.74, 117.03, 120.02, 120.63, 122.47, 125.17, 125.76, 127.30,

129.06, 129.75, 132.21, 133.53, 134.35, 134.65, 135.22, 135.80, 136.24, 136.91, 146.41, 166.40, 167.32, 168.83, 170.09, 171.14, 171.78, 172.81; HRMS (ESI): $C_{43}H_{43}Cl_2N_6O_9$ $[M + H]^+$: calcd 857.2469, found 857.2489.

***N*-(4-(3-(3-((2-(2,6-dioxopiperidin-3-yl)-1,3-dioxoisoindolin-4-yl)amino)propoxy)propoxy)butyl)-4-((2-(naphthalen-1-yl)-3,5-dioxo-1,2,4-thiadiazolidin-4-yl)methyl)benzamide (2).** Synthesized from **14** (0.112 g, 0.29 mmol), **15** (0.186 g, 0.27 mmol), DIPEA (0.42 mL, 0.313 g, 0.81 mmol), EDCI (0.204 g, 0.54 mmol), HOBT (0.039 g, 0.29 mmol). Obtained 0.110 g of yellow oil (48% yield). ^{1}H-NMR (400 MHz, DMSO) δ 1.52-1.53 (m, 5H), 1.71-1.81 (m, 5H), 1.97-2.01 (m, 2H), 2.81-2.99 (m, 2H), 3.27-3.44 (m, 10H), 4.92 (m, 2H), 5.00-5.04 (dd, 1H, J = 12.8, 5.6 Hz), 6.63 (t, 1H, *J* = 8.0 Hz), 6.98-7.0 (d, 1H, *J* = 8.0 Hz), 7.05-7.07 (d, 1H, *J* = 8.0 Hz), 7.45-7.47 (d, 1H, *J* = 8.0 Hz), 7.60-7.63 (m, 5H), 7.76-7.77 (d, 1H, *J* = 4.0 Hz), 7.82-7.84 (d, 2H, *J* = 8.0 Hz), 7.88-7.90 (m, 1H), 8.05-8.09 (m, 3H), 8.41 (t, 1H, *J* = 8.0 Hz), 11.06 (brs, 1H); ^{13}C-NMR (100 MHz, DMSO) δ 22.15, 25.98, 26.08, 28.87, 29.41, 30.97, 36.70, 45.34, 48.52, 67.84, 69.88, 70.04, 109.07, 110.36, 117.03, 122.39, 125.80, 126.85, 126.99, 127.22, 127.46, 127.54, 127.63, 127.91, 128.59, 130.08, 130.13, 131.04, 132.20, 133.99, 134.22, 136.23, 138.55, 146.40, 151.94, 165.79, 166.06, 167.30, 168.82, 170.07, 172.79; HRMS (ESI): $C_{43}H_{45}N_6O_9S$ $[M + H]^+$: calcd 821.9269, found 821.9281.

2-(3-(4-(2,4-dichlorophenyl)-2,5-dioxo-2,5-dihydro-1*H*-pyrrol-3-yl)-1*H*-indol-1-yl)-*N*-(2-(2-(2-((2-(2,6-dioxopiperidin-3-yl)-1,3-dioxoisoindolin-4-yl)amino)ethoxy)ethoxy)ethyl)acetamide (3). Synthesized from **8** (0.236 g, 0.97 mmol), **16** (0.394 g, 0.97 mmol), DIPEA (0.32 mL, 0.241 g, 1.87 mmol), EDCI (0.236 g, 1.24 mmol), HOBT (0.084 g, 0.62 mmol). Obtained 0.210 g of yellow oil (25% yield). ^{1}H-NMR (400 MHz, DMSO) δ 2.51-2.58 (m, 2H), 2.81-2.90 (m, 1H), 3.23-3.28 (m, 2H), 3.44-3.48 (m, 4H), 3.52-3.59 (m, 4H), 3.61-3.63 (m, 2H), 4.96 (s, 2H), 5.02-5.07 (dd, 1H, J = 14.0, 6.0 Hz), 6.35-6.37 (d, 1H, J = 8.0 Hz), 6.61 (t, 1H, J = 6.0 Hz), 6.76 (t, 1H, J = 7.6 Hz), 7.03-7.04 (d, 1H, J = 4.0 Hz), 7.08-7.15 (m, 2H), 7.35-7.39 (m, 2H), 7.46-7.48 (dd, 1H, J = 8.4, 2.0 Hz), 7-56-7.59 (m, 2H), 7.72 (d, 1H, J = 2.0 Hz), 8.12 (s, 1H), 8.39 (t, 1H, J = 6.0 Hz), 11.12 (m, 2H); ^{13}C-

NMR (100 MHz, DMSO) δ 22.12, 30.96, 41.69, 48.55, 48.96, 54.91, 68.89, 68.97, 69.61, 69.70, 104.35, 109.25, 110.69, 110.75, 117.43, 119.99, 120.62, 122.46, 125.16, 125.76, 127.30, 129.05, 129.74, 132.09, 133.53, 134.35, 134.64, 135.20, 135.81, 136.23, 136.90, 146.39, 166.62, 167.27, 168.95, 170.07, 172.79; HRMS (ESI): $C_{39}H_{35}Cl_2N_6O_9$ $[M + H]^+$: calcd 801.1843, found 801.1822.

***N*-(2-(2-(2-((2-(2,6-dioxopiperidin-3-yl)-1,3-dioxoisoindolin-4-yl)amino)ethoxy)ethoxy)ethyl)-4-((2-(naphthalen-1-yl)-3,5-dioxo-1,2,4-thiadiazolidin-4-yl)methyl)benzamide (4).** Synthesized from **14** (0.180 g, 0.46 mmol), **16** (0.191 g, 0.51 mmol), DIPEA (0.65 mL, 0.506 g, 0.92 mmol), EDCI (0.175 g, 0.92 mmol), HOBT (0.062 g, 0.46 mmol). Obtained 0.156 g of yellow oil (44% yield). ^{1}H-NMR (400 MHz, DMSO) δ 1.99-2.03 (m, 2H), 2.49-2.60 (m, 2H), 2.82-2.88 (m, 1H), 3.32-3.46 (m, 4H), 3.53-3.63 (m, 7H), 4.94 (s, 2H), 5.03-5.07 (dd, 1H, J = 14.0, 6.0 Hz), 6.60 (t, 1H, *J* = 8.0 Hz), 7.02-7.03 (d, 1H, *J* = 8.0 Hz), 7.10-7.12 (d, 1H, *J* = 8.0 Hz), 7.47-7.49 (d, 2H, *J* = 8.0 Hz), 7.54-7.67 (m, 4H), 7.79-7.92 (m, 4H), 8.07-8.11 (m, 2H), 8.50 (t, 1H, *J* = 8.0 Hz), 11.09 (brs, 1H); ^{13}C-NMR (100 MHz, DMSO) δ 22.13, 30.98, 41.69, 42.64, 45.35, 48.52, 48.60, 68.87, 68.93, 69.66, 109.25, 110.66, 117.41, 121.39, 122.41, 125.81, 126.86, 127.28. 127.48, 127.93, 128.35, 130.09, 130.14, 131.05, 132.08, 133.72, 133.99, 135.01, 136.21, 138.67, 143.59, 146.38, 151.95, 155.67, 165.92, 166.08, 167.29, 168.93, 170.08, 172.79; HRMS (ESI): $C_{39}H_{37}N_6O_9S$ $[M + H]^+$: calcd 765.2343, found 765.2331.

GSK-3β inhibition in vitro assay. Human recombinant GSK-3β, prephosphorylated polypeptide substrate and white 96-well plates were purchased from Millipore (Millipore Iberica S.A.U.). Kinase-Glo Luminescent Kinase Assay was obtained from Promega (Promega Biotech Iberica, SL). Adenosinetriphosphate (ATP) and all other reagents were from Sigma-Aldrich. Assay buffer contained 50 mM 4-(2-hydroxyethyl)-1- piperazineethanesulfonic acid (HEPES) (pH 7.5), 1 mM ethylenediaminetetraacetic acid (EDTA), 1 mM ethylene glycol tetraacetic acid (EGTA), and 15 mM magnesium acetate. The in solution experiments for GSK-3β inhibition analysis were performed by following the method developed by Baki et al.[1] In a typical assay, 10 μL of test compound (dissolved

in DMSO at 1 mM concentration and diluted in advance in assay buffer to the desired concentration) and 10 μL (20 ng) of enzyme were added to each well in presence of 20 μL of assay buffer containing GSM substrate and ATP in order to reach a 25 μM and 1 μM final concentration respectively. The final DMSO percentage in the reaction mixture did not exceed 1%. After a 30 min incubation time at 37 °C, the enzymatic reaction was stopped by adding 40 μL of Kinase-Glo reagent. Glow-type luminescence was recorded after 10 min using a Victorx3 (PerkinElmer Waltham, MA, USA) plate reader. The activity was found proportional to the difference of the total and consumed ATP. The inhibitory activities were calculated based on maximal kinase (average positive) and luciferase activities (average negative) measured in the absence of inhibitor and in the presence of reference compound inhibitor (SB-415286 Merck Millipore, IC_{50} = 55 nM) at total inhibition concentration (5 μM), respectively. The linear regression parameters were determined and the IC_{50} extrapolated by GraphPad software (version 5.0; GraphPad Software, La Jolla, CA, USA).

8. Copies of ^{1}H-NMR and ^{13}C-NMR spectra.

Compound 1. ^{1}H-NMR (400 MHz) and ^{13}C-NMR (100 MHz) in DMSO-d

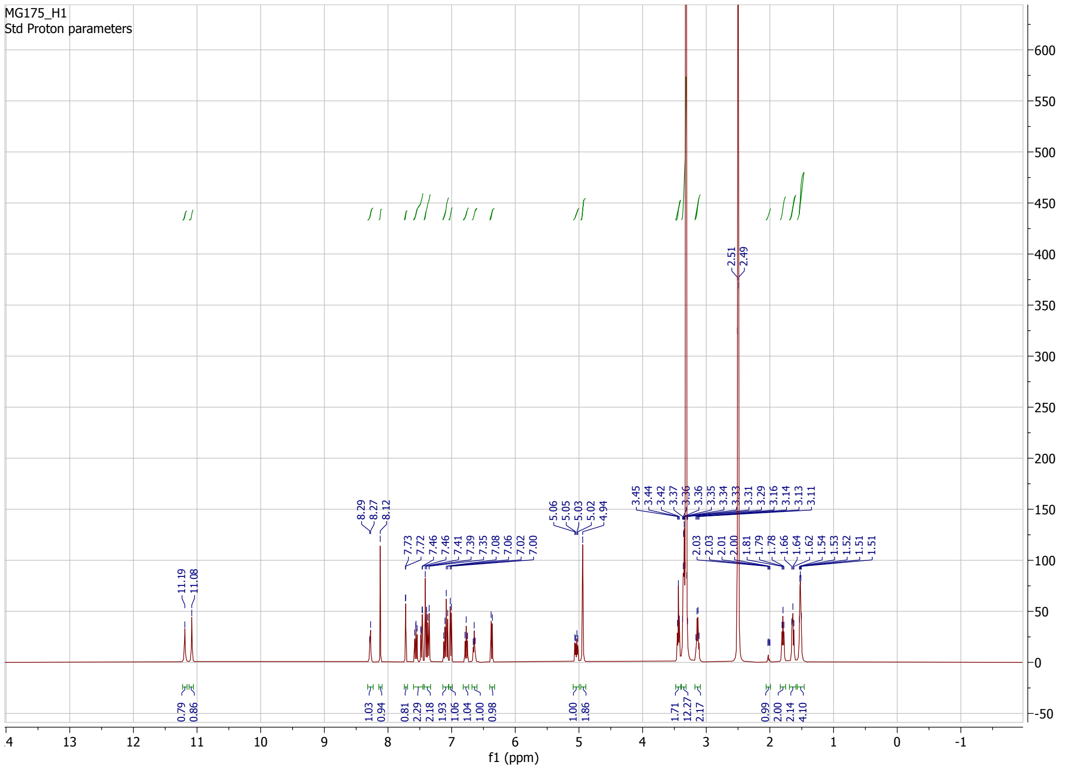

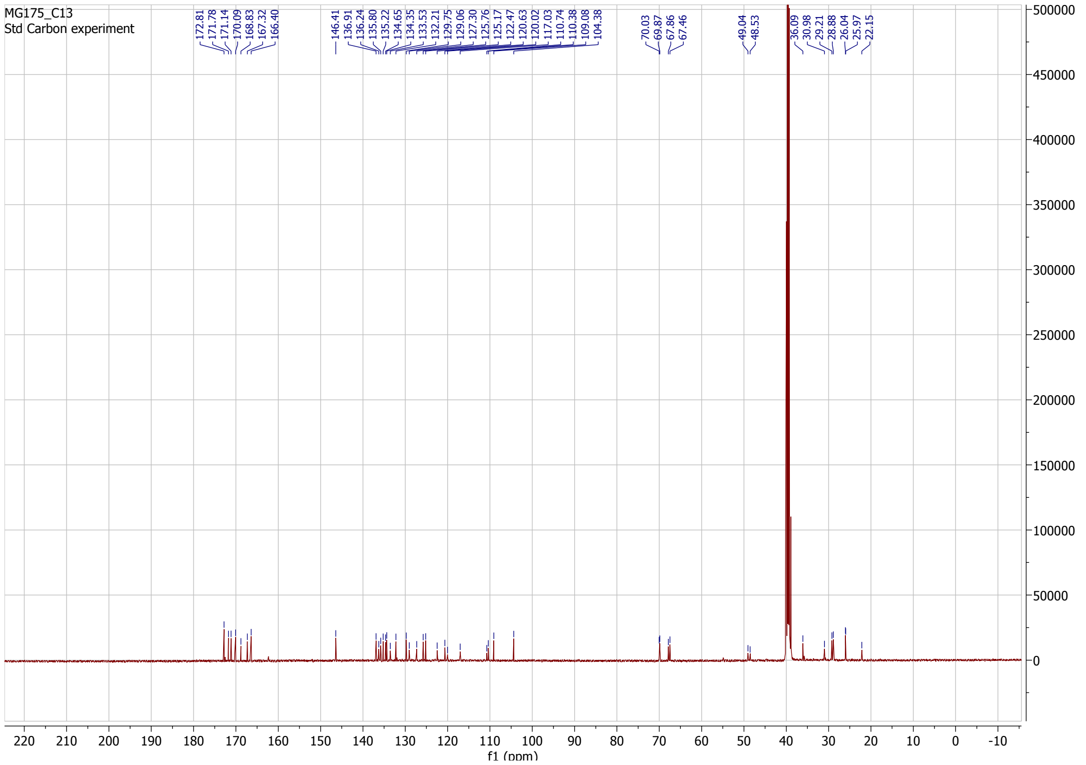

Compound 2. ^{1}H-NMR (400 MHz) and ^{13}C-NMR (100 MHz) in DMSO-d

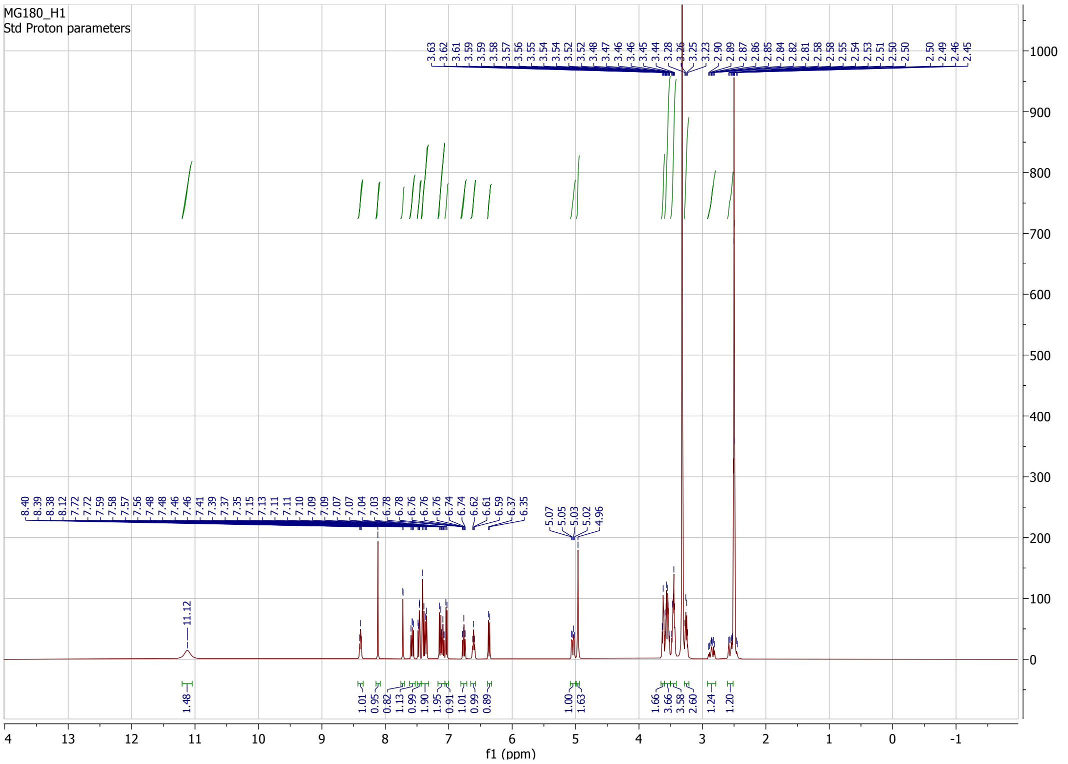

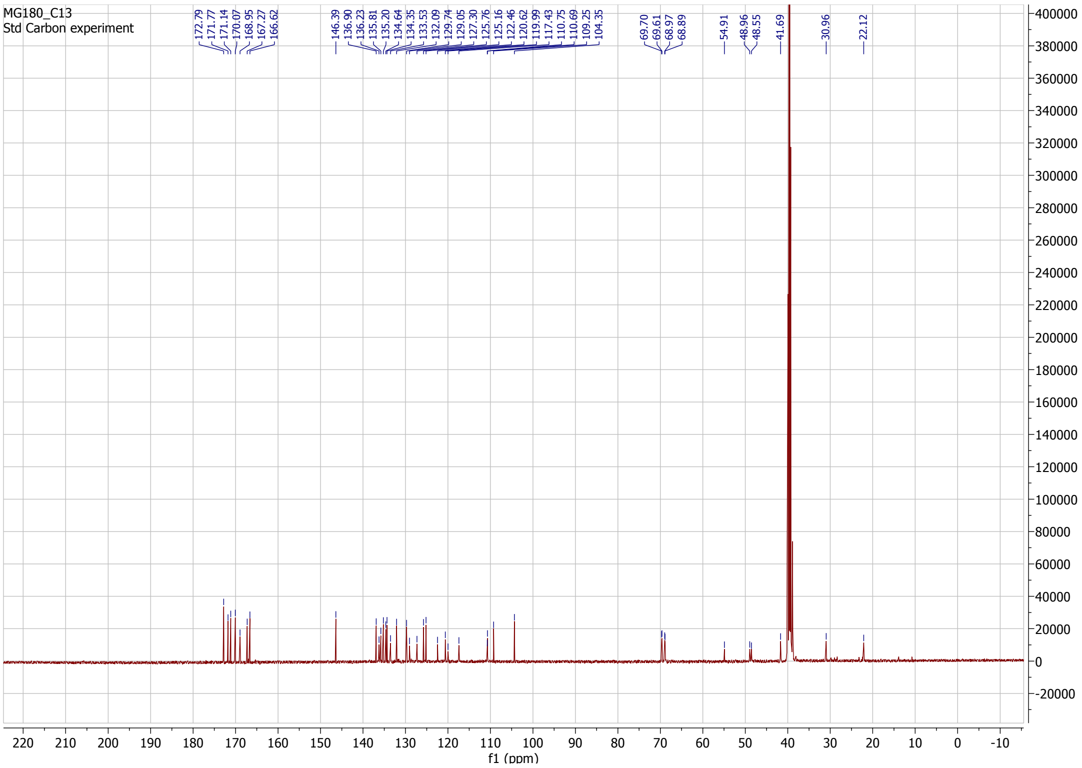

Compound 3. ^{1}H-NMR (400 MHz) and ^{13}C-NMR (100 MHz) in DMSO-d

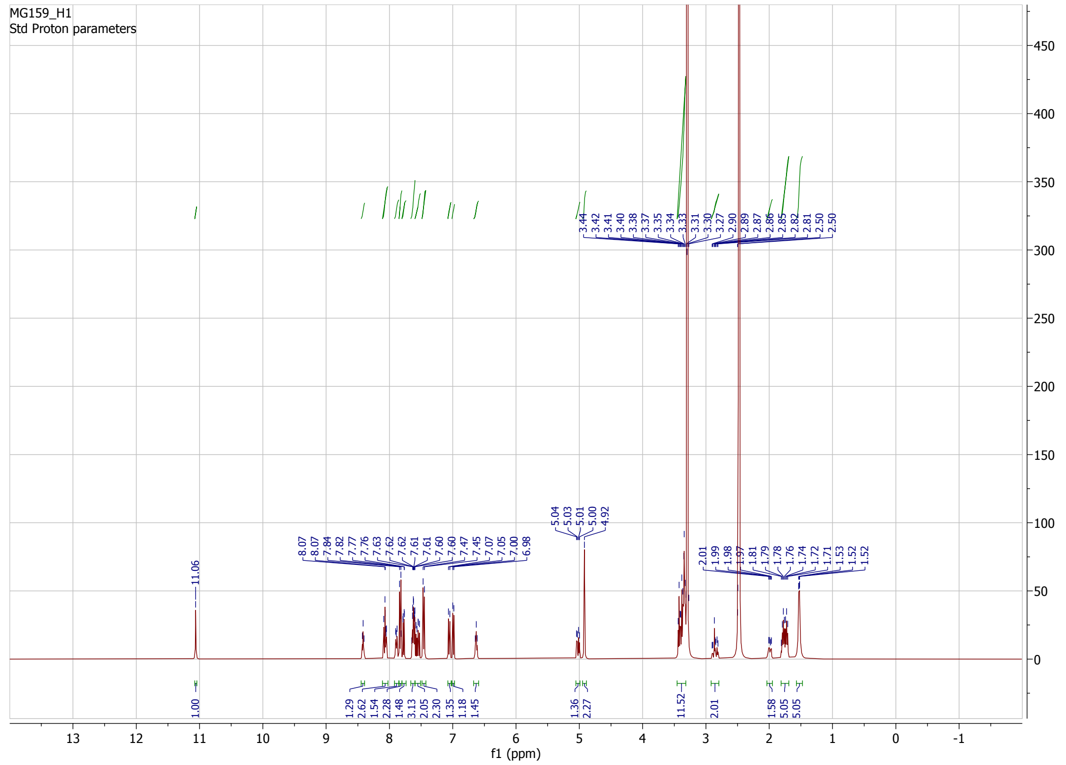

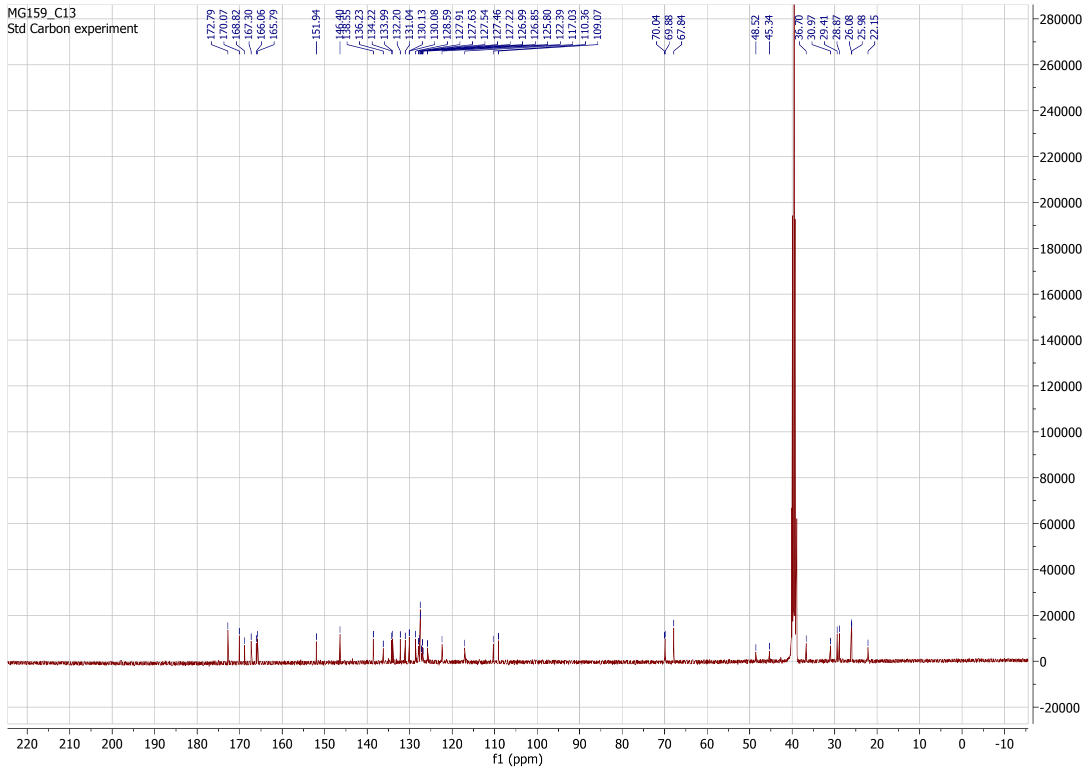

Compound 4. ^{1}H-NMR (400 MHz) and ^{13}C-NMR (100 MHz) in DMSO-d

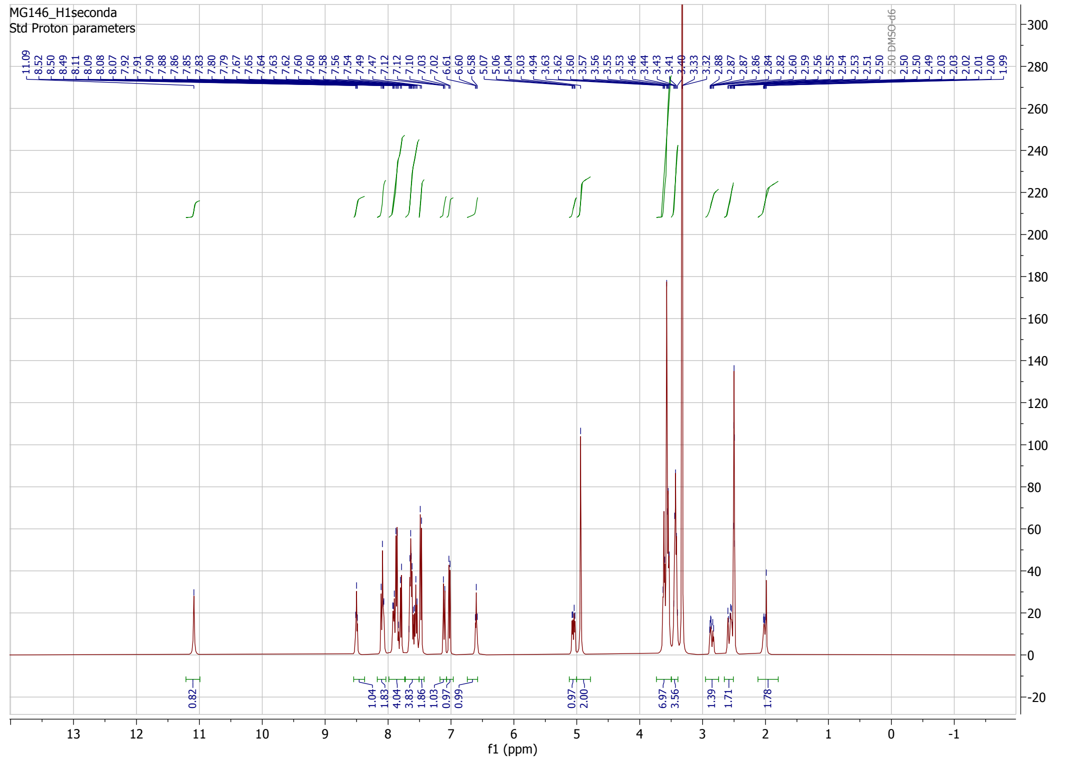

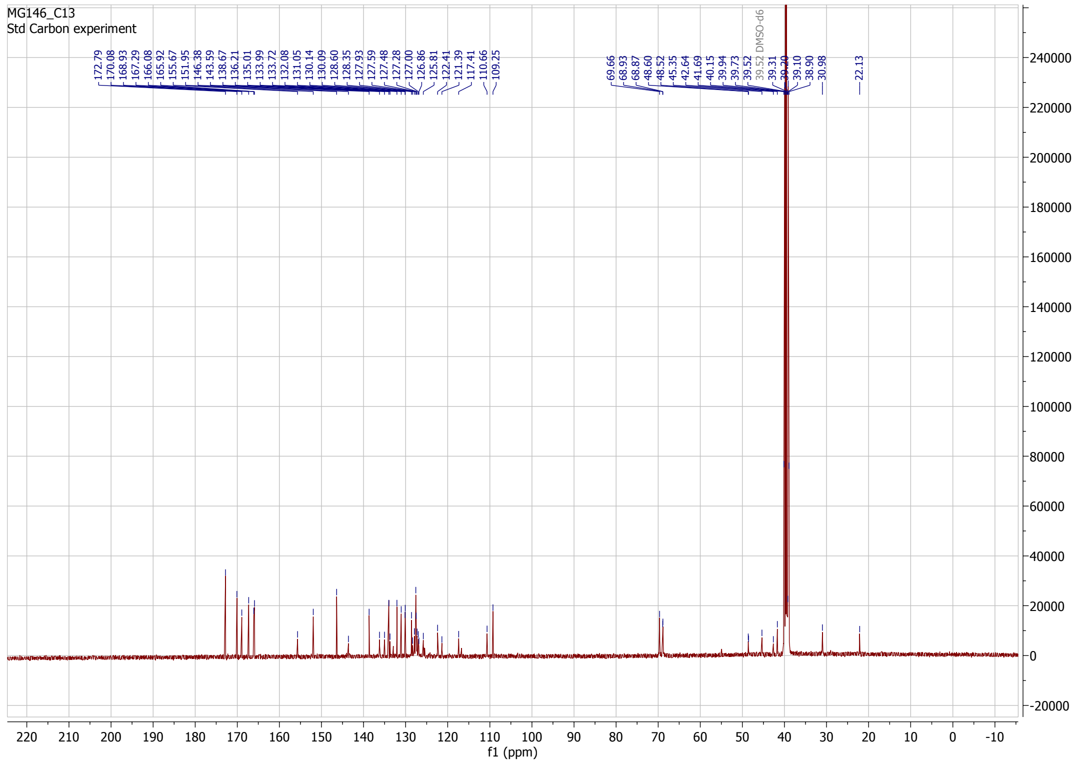

9. Computational Analysis

Docking study. The monomeric structure of human GSK-3β co-crystallized with an ATP-competitive inhibitor was retrieved from the Protein Data Bank (PDB ID: 1q4l). Compounds 1 and 3 were docked in 1q4l ATP binding site using GOLD (release 5.5).[2] For obtaining the two GSK-3β-ligand complexes, a 25Å radius sphere centered on Ala83 β-carbon was chosen as the region to perform docking simulations. In the docking studies the key bridging water between Thr138 and Gln185.[3] was retained, and 30 poses were generated for each ligand. Two H-bond constraints between maleimide inhibitor ring and the backbone of Asp133 and Val135 were set to maintain the classic maleimide inhibitor binding mode,[4] and to orient the compound linker and the pomalidomide moiety towards the β-hairpin loop made up by residues 74-80. This loop was supposed to be the key for the E3 ligase cereblon (CRBN) protein binding, as shown by previous structural studies performed on casein kinase CK-1α in complex with CRBN and lenalidomide (PDB ID: 5fqd).[2]

Molecular Dynamics. Starting from docking results, a GSK-3β-ligand complex with the pomalidomide moiety directed towards the kinase β-hairpin loop region was selected for each ligand. MG175 and MG180 were parametrized through resp method, and the two complexes were solvated using ambertools in BiKi (release 1.5).[5] The solvated protein-ligand systems were equilibrated following three steps of NVT thermalization of 0,1 ns and a final NPT production step of 1ns, constraining ligand and backbone positions in the first two steps. The prepared complexes were then subjected to two replicas of 200 ns molecular dynamic (MD) simulation, to evaluate the different behavior of the compounds according to the variation of the linker chain length. In all MD simulation, ff99SB-ILDN AMBER force-field[6] and GROMACS engine[7] were adopted. Distances between ligand pomalidomide moiety and kinase loop Lys74 and Gly79 interacting atoms were determined along the four obtained trajectories.

Linker analysis. Compounds **1-4** were parametrized through resp method and were solvated using amber tools in BiKi (release 1.5, 10.1021/acs.jcim.7b00680). The solvated ligand systems were

equilibrated following three steps of NVT thermalization of 0,1 ns and a final NPT production step of 1ns, constraining ligand atom positions in the first two steps. The prepared complexes were then subjected to two replicas of 200 ns molecular dynamic (MD) simulation, to evaluate the different behavior of the compounds in water according to the variation of the linker chain length. In all MD simulation, ff99SB-ILDN AMBER force-field (10.1002/prot.22711) and GROMACS engine (10.1016/j.softx.2015.06.001) were adopted. (Molecular Dynamics (MD) workflow adopted was identical to which used for the 200ns MD GSK-PROTAC complexes). Total, hydrophobic and hydrophilic SASA, as well as the radius of gyration, were calculated along the MD trajectories using GROMACS 5.1 tools. The number of H-bonds (Hb) and of donor-acceptor pairs (DAp) was also calculated with GROMACS 5.1 tools. To determine if an H-bond exists, two geometrical criterions were used: donor-acceptor distance and acceptor-donor-hydrogen angle must be lower than 0.35 nm and 30°, respectively.

Every 100 frames (1ns) the moving means of the total, hydrophobic and hydrophilic SASA, of Hb and Dap values were calculated and plotted using MATLAB.

10. Biology

Cell Cultures

Human neuronal SH-SY5Y cells (Sigma-Aldrich, St. Louis, MO, USA) were routinely grown in Dulbecco's modified Eagle's Medium supplemented with 10% fetal bovine serum, 2 mM l-glutamine, 50 U/mL penicillin, and 50 μg/mL streptomycin at 37 °C in a humidified incubator with 5% CO_2.

Determination of cytotoxicity

SH-SY5Y cells were seeded in a 96-well plate at 2×10^4 cells/well, incubated for 24 h and then treated with different concentrations (1.25 – 40 μM) of compounds **1-4**, SB-216763 and tideglusib for 24 h at 37°C in 5% CO_2. The cell viability, in terms of mitochondrial metabolic function, was evaluated by the reduction of 3-(4,5-dimethyl-2-thiazolyl)-2,5-diphenyl-2H-tetrazolium bromide

(MTT) to its insoluble formazan. Briefly, the treatment was replaced with MTT in HBSS [0.5 mg/mL] for 2 h at 37°C in 5% CO_2. After washing with HBSS, formazan crystals were dissolved in isopropanol. The amount of formazan was measured (570 nm, reference filter 690 nm) using a multilabel plate reader (VICTOR™ X3, PerkinElmer, Waltham, MA, USA).[8] Data are expressed as mean of three independent experiments.

Determination of GSK-3β degradation by Western Blotting

SH-SY5Y cells were seeded in 60 mm dishes at 2×10^6 cells/dish, incubated for 24 h, and then treated with compounds **1-4**, SB-216763 and tideglusib at the concentration of 10 µM for 48 h at 37 °C in 5% CO_2. Cells were also treated with compound 1 at different concentrations (0.5 – 1 – 5 – 10 µM) for 48 h or for 24 h in the presence of lactacystin (1.25 – 2.5 – 5 µM). At the end of incubation, cells were trypsinized and the cellular pellet was resuspended in complete lysis buffer containing leupeptin (2 µg/mL, Sigma-Aldrich), phenylmethylsulfonyl fluoride (PMSF, 100 µg/mL, Sigma-Aldrich) and a cocktail of protease/phosphatase inhibitors (100×). The protein concentration was determined using the Bradford method (Bio-Rad Laboratories Srl, Hercules, CA, USA). The protein lysates (50 µg per sample) were separated by Mini-PROTEAN TGX Stain-Free™ precast gels (12% SDS polyacrylamide gels, Bio-Rad Laboratories SrL) and electroblotted onto 0.45 µm nitrocellulose membranes. Membranes were incubated overnight at 4 °C with primary antibody recognizing GSK-3β (1:1000, Cell Signaling Technologies Inc, Danvers, MA, USA). Membranes were then washed with TRIS-buffered saline-T (TBS + 0.05% Tween20), and then incubated with horseradish peroxidase (POD) linked anti-rabbit secondary antibody (1:2000, GE Healthcare, Chicago, IL, USA). Immunoreactive bands were visualized by enhanced chemiluminescence (ECL, Bio-Rad Laboratories Srl). The same membranes were stripped and reprobed with anti β-actin (1:1000, Sigma-Aldrich). Data were normalized on the β-actin bands and analyzed by densitometry, using the Quantity One software (Bio-Rad Laboratories Srl).[9] Data are reported as mean ± SEM of three independent experiments.

Determination of neuroprotective effects against $CuSO_4$

SH-SY5Y cells were seeded in a 96-well plate at 2 × 10^4 cells/well, incubated for 24 h and then treated with compound **1** (0.5 – 1 μM), SB-216763 (1 μM) and tideglusib (1 μM) for 24 h in the presence of $CuSO_4$ (150 μM) at 37 °C in 5 % CO_2. Cells were also treated with compound **1** (1 μM) for 24 h in the presence of lactacystin (5 μM). At the end of the treatment, the cell viability, in terms of mitochondrial metabolic function, was evaluated by the reduction of 3-(4,5-dimethyl-2-thiazolyl)-2,5-diphenyl-2H-tetrazolium bromide (MTT) to its insoluble formazan. Briefly, the treatment was replaced with MTT in HBSS [0.5 mg/mL] for 2 h at 37°C in 5% CO_2. After washing with HBSS, formazan crystals were dissolved in isopropanol. The amount of formazan was measured (570 nm, reference filter 690 nm) using a multilabel plate reader (VICTOR™ X3, PerkinElmer, Waltham, MA, USA).[10] Data are reported as mean ± SEM of three independent experiments. The neurotoxicity was expressed as a percentage of control cells and calculated by the formula: 100 – ((absorbance of treated neurons/absorbance of untreated neurons) × 100)

Determination of neuroprotective effects against $A\beta_{25\text{-}35}$ *peptide*

$A\beta_{25\text{-}35}$ peptide preparation for neurotoxicity assay. $A\beta_{25\text{-}35}$ peptide was dissolved in hexafluoroisopropanol (HFIP) to 1 mg/mL, sonicated and incubated at room temperature for 24 h to produce unaggregated Aβ peptide. The HFIP was dried under vacuum in a Speed Vac and the resulting peptide film was dissolved in DMSO to 1 mM. The unaggregated $A\beta_{25\text{-}35}$ stock solution was then aliquoted and stored at -20 °C until use. For neurotoxicity assay, the $A\beta_{25\text{-}35}$ stock solution was diluted directly into cell culture media.

Neurotoxicity assay. SH-SY5Y cells were seeded in a 96-well plate at 3 × 10^4 cells/well, incubated for 24 h and then treated with different concentrations (0.5 – 1 μM) of compound 1 for 2 h and subsequently for 3 h in the presence of unaggregated $A\beta_{25\text{-}35}$ peptide (10 μM) at 37 °C in 5 % CO_2. The cell viability, in terms of mitochondrial metabolic function, was evaluated by the reduction of 3-(4,5-dimethyl-2-thiazolyl)-2,5-diphenyl-2H-tetrazolium bromide (MTT) to its insoluble formazan. Briefly, the treatment was replaced with MTT in HBSS [0.5 mg/mL] for 2 h at 37°C in 5% CO_2. After washing with HBSS, formazan crystals were dissolved in isopropanol. The amount of formazan was

measured (570 nm, reference filter 690 nm) using a multilabel plate reader (VICTOR™ X3, PerkinElmer, Waltham, MA, USA).[11] Data are reported as mean ± SEM of three independent experiments. The neurotoxicity was expressed as a percentage of control cells and calculated by the formula: 100 – ((absorbance of treated neurons/absorbance of untreated neurons) × 100).

Statistical analysis

Results are shown as mean ± standard error (SEM) of three independent experiments. Statistical analysis was performed using one-way ANOVA with the Dunnett post hoc test. Differences were considered significant at $p < 0.05$. Analyses were performed using GraphPad PRISM software (version 5.0; GraphPad Software, La Jolla, CA, USA) on a Windows platform.

11. PAMPA BBB permeation studies.

Prediction of the brain penetration was evaluated using a parallel artificial membrane permeability assay (PAMPA).[12] Ten commercial drugs, PBS at pH 7.4, EtOH, and dodecane were purchased from Sigma- Aldrich, Acros organics, Merck, and Fluka. The porcine polar brain lipid (PBL) was from Avanti Polar Lipids. The donor plate was a 96-well filtrate plate (Multiscreen® IP Sterile Plate PDVF membrane, pore size is 0.45 μM) and the acceptor plate was an indented 96-well plate (Multiscreen®) both from Millipore. Filter PDVF membrane units (diameter 30 mm, pore size 0.45 μm) from Symta were used to filter the samples. A 96-well plate UV reader (Biteck Instruments, Synergy H4 Hibrid Reader) was used for the UV measurements. Ten compounds (3-5 mg of enoxacin, caffeine, desipramine, piroxicam, ofloxacin, hydrocortisone, beta-Estradiol, norfloxacin and testosterone; and 12 mg of promazine) were dissolved in 1 mL of EtOH. 100 μL of this compound stock solution were taken and 1400 μL of EtOH and 3500 μL of PBS were added to reach 30% of EtOH concentration in the experiment. These solutions were filtered. The acceptor 96-well microplate was filled with 180 μL of PBS/EtOH (70/30). The donor 96-well plate was coated with 4 μL of porcine brain lipid in dodecane (20 mg mL^{-1}) and after 5 min, 180 μL of each compound solution was added. 1 mg of compounds **1** and **3** to be determined their ability to pass the brain barrier were dissolved in 0.6 mL

of EtOH and 1.4 mL of PBS, filtered and then added to the donor 96-well plate. Then the donor plate was carefully put on the acceptor plate to form a “sandwich”, which was left undisturbed for 2 h and 45 min at 25 °C. During this time the compounds diffused from the donor plate through the brain lipid membrane into the acceptor plate. After incubation, the donor plate was removed. UV plate reader determined the concentration of compounds and commercial drugs in the acceptor and the donor plates. Every sample was analyzed at three to five wavelengths (between 200 and 500 nm), in 3 wells and in two independent runs. Results are given as the mean and the average of the two runs is reported. Quality control compounds (previously mentioned) of known BBB permeability were included in each experiment to validate the analysis set. A good correlation between experimental-described values was obtained P_e (exptl)= 2.3307 (bibl) + 6.4534 (R^2 = 0.9818) (Figure 7SI). From this equation and following the pattern established in the literature for BBB permeation prediction, we could classify compounds as CNS + when they present a permeability > 15.78 x 10^{-6} cm s^{-1}.

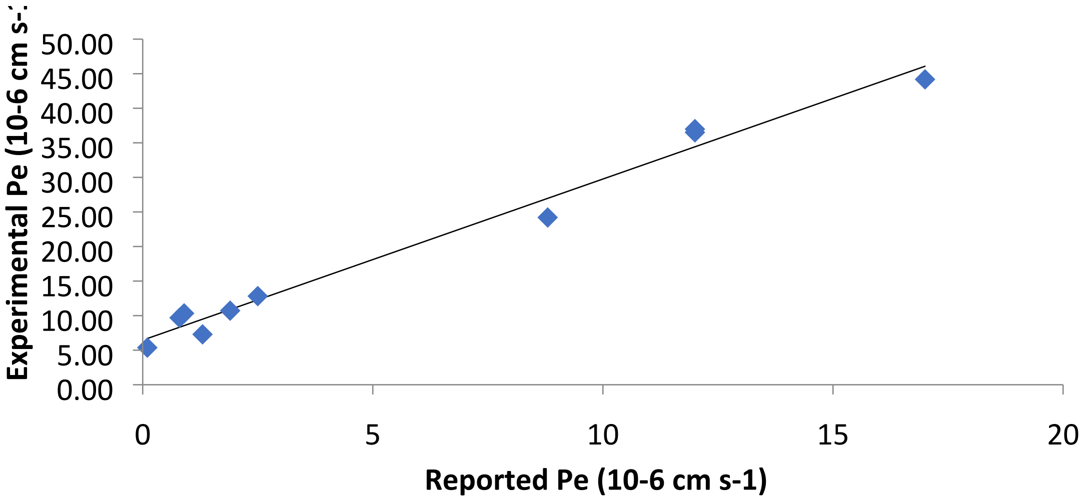

Figure 7SI. Linear correlation between experimental and reported permeability of commercial drugs using the PAMPA-BBB assay.

Table 1SI. Permeability (P_e: 10^{-6} cm s^{-1}) in the PAMPA-BBB assay for the commercial drugs (used in the validation study) and compounds **1** and **3** with their predictive penetration in the CNS.

Compound	P_e (literature data)[b]	P_e (experimental data)[c]	Prediction
Caffeine	1.3	7.29 ± 0.57	
Desipramine	12	36.51 ± 5.35	
Enoxacin	0.9	10.33 ± 0.95	
Hydrocortisone	1.9	10.72 ± 1.68	
Ofloxacin	0.8	9.69 ± 1.12	
Piroxicam	2.5	12.82 ± 0.63	
Promazine	8.8	24.20 ± 0.89	
Testosterone	17	44.18 ± 2.53	
beta-estradiol	12	36.98 ± 2.19	
Norfloxacin	0.1	5.37 ± 0.17	
**1**		15.33 ± 1.12	CNS +/-
**3**		20.68 ± 3.93	CNS +

[a]PBS:EtOH (70:30) was used as solvent; [b]Ref. [12]; [c] Data are the mean ± SD of two independent experiments.

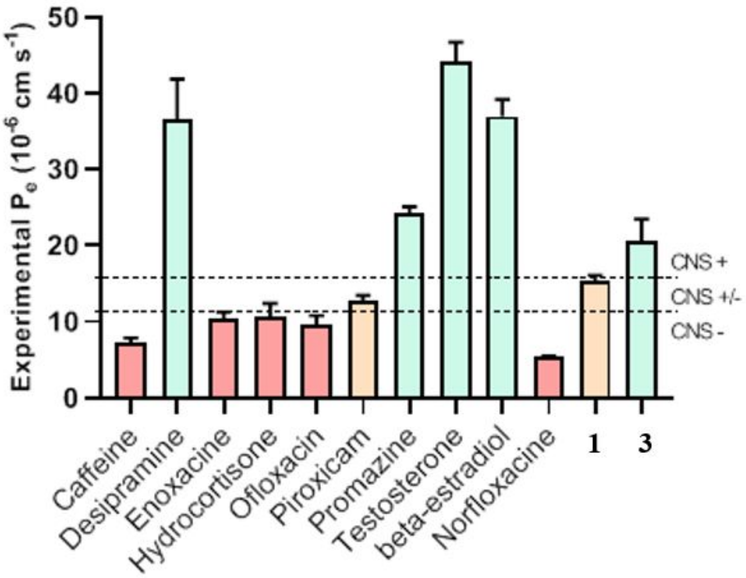

Figure 8SI. PAMPA in vitro permeability (P_e) plot of compounds **1** and **3** and the reference drugs. CNS + (green) for $P_e > 15.78 \times 10^{-6}$ cm s^{-1}, CNS- (red) for compounds with $P_e < 11.11 \times 10^{-6}$ cm $^{s-1}$, CNS +/- (orange) for 15.78×10^{-6} cm $s^{-1} > P_e > 11.11 \times 10^{-6}$ cm $^{s-1}$. Mean ± SD from two independent experiments is represented.

12. References

[1] A. Baki, A. Bielik, L. Molnár, G. Szendrei, G. M. Keserü, *Assay Drug Dev Technol* **2007**, *5*, 75-83.

[2] G. Petzold, E. S. Fischer, N. H. Thomä, *Nature* **2016**, *532*, 127-130.

[3] S. Y. Lu, Y. J. Jiang, J. Lv, J. W. Zou, T. X. Wu, *J Comput Chem* **2011**, *32*, 1907-1918.

[4] T. Kramer, B. Schmidt, F. Lo Monte, *Int J Alzheimers Dis* **2012**, *2012*, 381029.

[5] S. Decherchi, G. Bottegoni, A. Spitaleri, W. Rocchia, A. Cavalli, *J Chem Inf Model* **2018**, *58*, 219-224.

[6] K. Lindorff-Larsen, S. Piana, K. Palmo, P. Maragakis, J. L. Klepeis, R. O. Dror, D. E. Shaw, *Proteins* **2010**, *78*, 1950-1958.

[7] S. Pronk, S. Páll, R. Schulz, P. Larsson, P. Bjelkmar, R. Apostolov, M. R. Shirts, J. C. Smith, P. M. Kasson, D. van der Spoel, B. Hess, E. Lindahl, *Bioinformatics* **2013**, *29*, 845-854.

[8] C. J. Cristancho Ortiz, M. de Freitas Silva, L. Pruccoli, N. Fonseca Nadur, L. L. de Azevedo, A. E. Kümmerle, I. A. Guedes, L. E. Dardenne, L. F. Leomil Coelho, M. J. Guimarães, F. M. R. da Silva, N. Castro, V. S. Gontijo, V. C. T. Rojas, M. K. de Oliveira, F. C. Vilela, A. Giusti-Paiva, G. Barbosa, L. M. Lima, G. B. Pinheiro, L. G. Veras, M. R. Mortari, A. Tarozzi, C. Viegas, *RSC Med Chem* **2022**, *13*, 568-584.

[9] L. Pruccoli, F. Morroni, G. Sita, P. Hrelia, A. Tarozzi, *Antioxidants (Basel)* **2020**, *9*.

[10] A. De Simone, V. La Pietra, N. Betari, N. Petragnani, M. Conte, S. Daniele, D. Pietrobono, C. Martini, S. Petralla, R. Casadei, L. Davani, F. Frabetti, P. Russomanno, E. Novellino, S. Montanari, V. Tumiatti, P. Ballerini, F. Sarno, A. Nebbioso, L. Altucci, B. Monti, V. Andrisano, A. Milelli, *ACS Med Chem Lett* **2019**, *10*, 469-474.

[11] A. Tarozzi, M. Bartolini, L. Piazzi, L. Valgimigli, R. Amorati, C. Bolondi, A. Djemil, F. Mancini, V. Andrisano, A. Rampa, *Pharmacol Res Perspect* **2014**, *2*, e00023.

[12] L. Di, E. H. Kerns, K. Fan, O. J. McConnell, G. T. Carter, *Eur J Med Chem* **2003**, *38*, 223-232.
